# Supplementary material for: Programmable mutually exclusive alternative splicing for generating RNA and protein diversity
Source: Nat Commun. 2019 Jun 17;10:2673. doi: 10.1038/s41467-019-10403-w (PMC6572816; doi:10.1038/s41467-019-10403-w)
Supplement: Supplementary file 1 — Supplementary Information [file 41467_2019_10403_MOESM1_ESM.pdf]

SUPPLEMENTARY INFORMATION

Programmable mutually exclusive alternative splicing for generating RNA and protein diversity

Mathur, et al.

SUPPLEMENTARY INFORMATION

Supplementary Figure 1. Fluorescence analysis of a splice site sequence mutation in the ASD mCherry controls.

Supplementary Figure 2. Fluorescence analysis of splice site sequence mutations in the ASD Clover controls.

Supplementary Figure 3. Regulatory sequence profiles of mutually exclusive exons in ASD mCherry and ASD Clover.

Supplementary Figure 4. Characterization of exon length flexibility in the MEAS intron framework.

Supplementary Figure 5. Producing alternate isoform profiles with ASD Clover by tuning consensus sequence element strengths.

Supplementary Figure 6. Fluorescence microscopy images of ASD mCherry devices in HEK-293T cells.

Supplementary Figure 7. Assessment of ASD mCherry devices in HeLa, CHO-K1, and U2OS cells.

Supplementary Figure 8. Regulatory sequence profiles of mutually exclusive exons in ASD TALE-TFs.

Supplementary Figure 9. RNA isoform profiles from ASD TALE-TF 1 T/G devices.

Supplementary Figure 10. Fluorescence microscopy images of ASD TALE-TF 1 T/G devices in HEK-293T cells.

Supplementary Figure 11. Spliced transcription factors activate gene expression from swapped promoter binding sites.

Supplementary Figure 12. Gene activation with ASD TALE-TF 1 T/A from promoter binding sites harboring a one-nucleotide mismatch.

Supplementary Figure 13. RNA isoform profiles for ASD TALE-TF 2 TT/AA devices.

Supplementary Figure 14. Gene activation with ASD TALE-TF 2 TT/GG from promoter binding sites harboring a two-nucleotide mismatch.

Supplementary Figure 15. Plasmid maps.

Supplementary Figure 16. Representative flow cytometry plots exemplifying the gating strategy for ASD mCherry.

Supplementary Figure 17. Representative flow cytometry plots exemplifying the gating strategy for ASD TALE-TF 1 T/G.

Supplementary Table 1. List of plasmids used in this study.

Supplementary Table 2. List of representative primer sequences used in this study.

Supplementary Table 3. List of intron sequences used in this study.

Supplementary Table 4. List of exon sequences used in this study.

Supplementary References

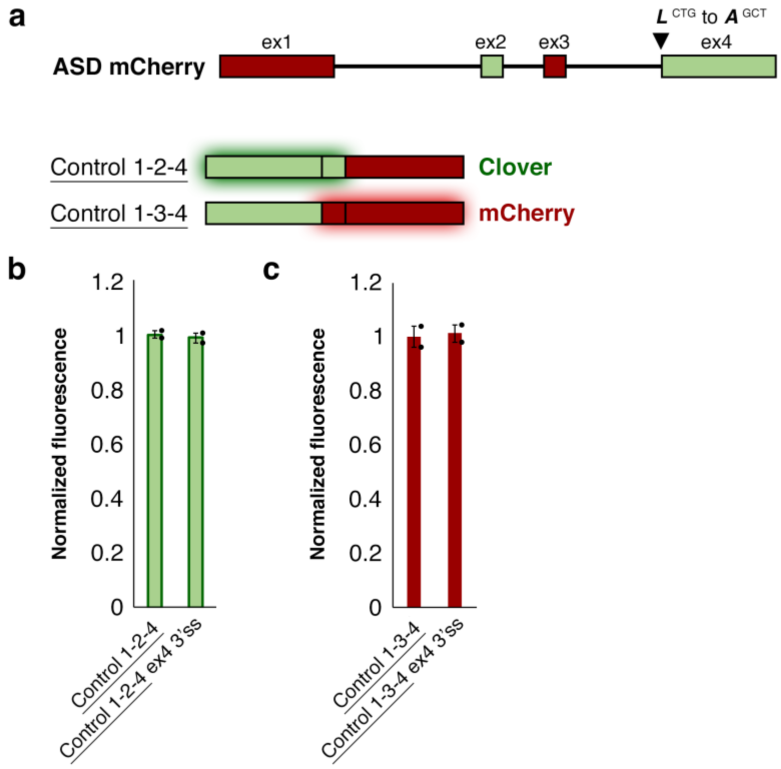

Supplementary Figure 1. Fluorescence analysis of a splice site sequence mutation in the ASD mCherry controls. (a) Schematic illustrating the nucleotide changes for incorporating a splice site sequence from the intron framework into an exon of ASD mCherry. A specific codon was set to maintain splice site sequence and its corresponding amino acid is shown in italicized bold. Control 1-2-4 expresses Clover and control 1-3-4 expresses mCherry, and neither harbors any mutations. (b) Clover fluorescence from control 1-2-4 and its variant that contains a splice site sequence mutation. (c) mCherry fluorescence from control 1-3-4 and its variant that contains a splice site sequence mutation. Fluorescence from the controls and their variants was quantified via flow cytometry. The median fluorescence of each population was measured and normalized to the median fluorescence intensities of the BFP transfection marker and its corresponding control 1-2-4 or control 1-3-4. Median values from biological duplicates were averaged and reported with an error range of ±1 standard deviation. Source data are provided as a Source Data file.

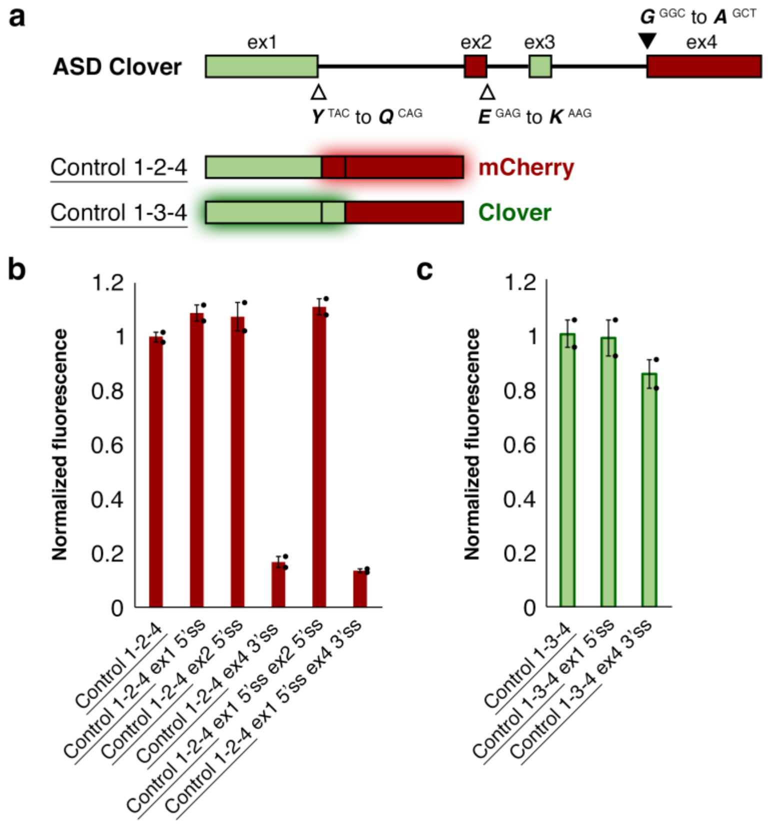

Supplementary Figure 2. Fluorescence analysis of splice site sequence mutations in the ASD Clover controls. (a) Schematic illustrating the nucleotide changes for incorporating splice site sequences from the intron framework into the exons of ASD Clover. Specific codons were set to maintain splice site sequences and their corresponding amino acids are shown in italicized bold. Control 1-2-4 expresses mCherry and control 1-3-4 expresses Clover, and neither harbors any mutations. (b) mCherry fluorescence from control 1-2-4 and its variants that contain splice site sequence mutations. (c) Clover fluorescence from control 1-3-4 and its variants that contain splice site sequence mutations. Fluorescence from the controls and their variants was quantified via flow cytometry. The median fluorescence of each population was measured and normalized to the median fluorescence intensities of the BFP transfection marker and its corresponding control 1-2-4 or control 1-3-4. Median values from biological duplicates were averaged and reported with an error range of ±1 standard deviation. Source data are provided as a Source Data file.

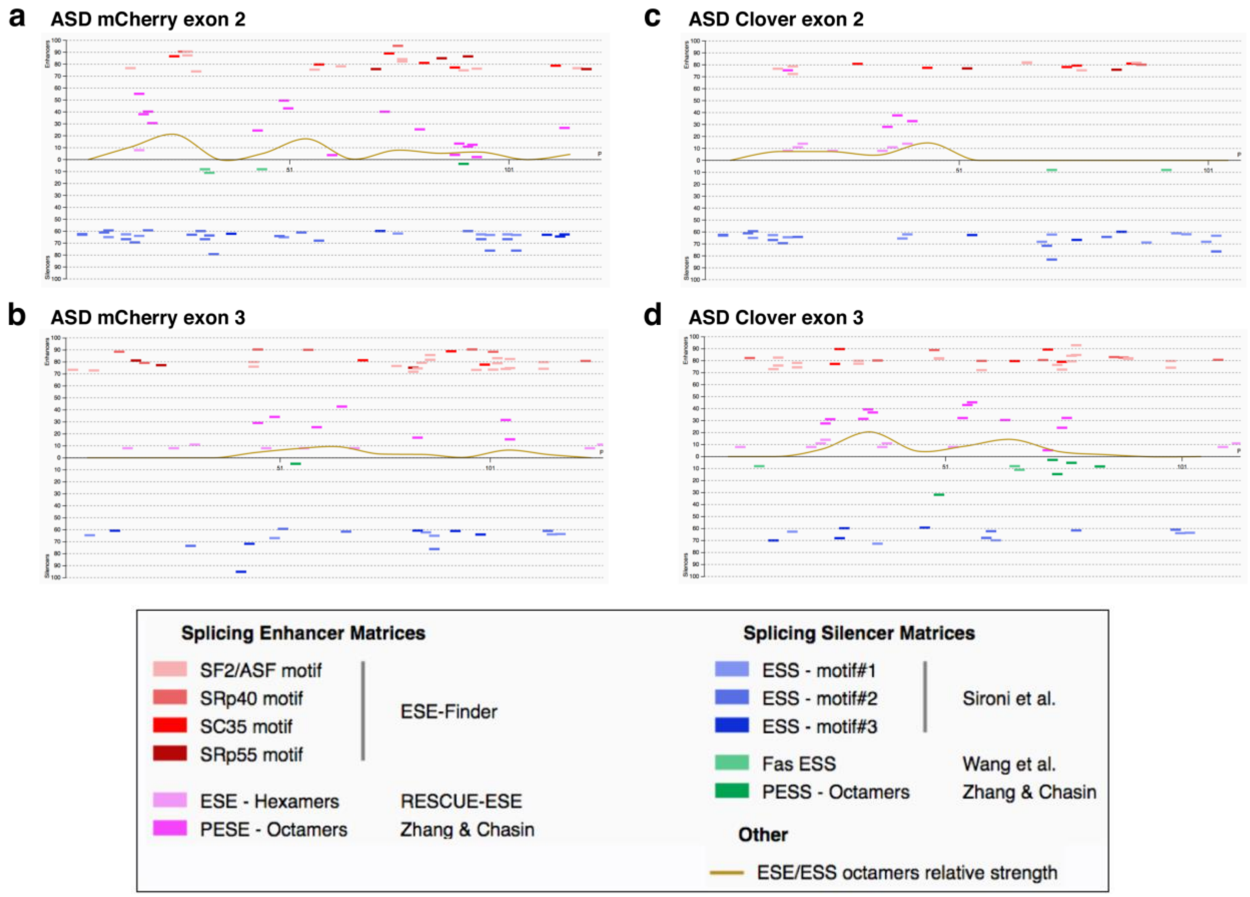

Supplementary Figure 3. Regulatory sequence profiles of mutually exclusive exons in ASD mCherry and ASD Clover. The ESE/ESS profiles for (a) ASD mCherry exon 2, (b) ASD mCherry exon 3, (c) ASD Clover exon 2, and (d) ASD Clover exon 3. Enhancer motifs are colored in shades of red and pink. Silencer motifs are colored in shades of blue and green. Each color corresponds to motifs from a different prediction algorithm as described in the legend. The yellow line indicates relative ESE/ESS strength.

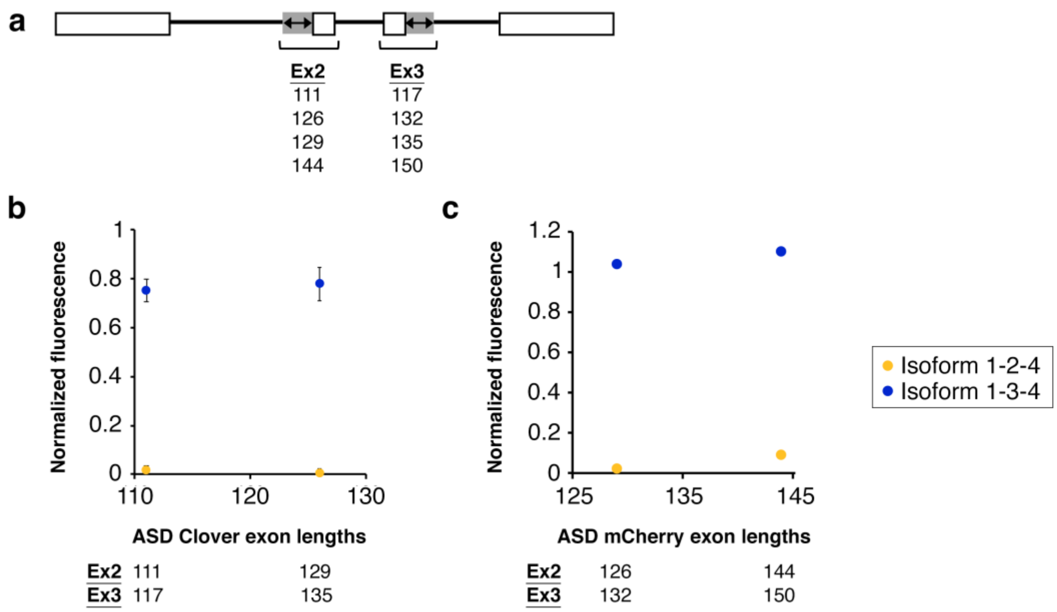

Supplementary Figure 4. Characterization of exon length flexibility in the MEAS intron framework. (a) Mutually exclusive exon sequence lengths were extended from their base lengths by adding linker sequences (Gly-Ser-Ser-Gly-Ser-Ser) with the nucleotide sequence of GGCTCCTCCGGCTCCTCC to exons 2 and 3. (b) Isoform 1-2-4 (mCherry fluorescence) and isoform 1-3-4 (Clover fluorescence) levels from a systematic assessment of mutually exclusive exon lengths in ASD Clover. (c) Isoform 1-2-4 (Clover fluorescence) and isoform 1-3-4 (mCherry fluorescence) levels from a systematic assessment of mutually exclusive exon lengths in ASD mCherry. Fluorescence from the splicing devices was quantified via flow cytometry. The median fluorescence of each population was measured and normalized to the median fluorescence intensities of the BFP transfection marker and the spliced controls. Median values from biological duplicates were averaged and reported with an error range of ±1 standard deviation. Source data are provided as a Source Data file.

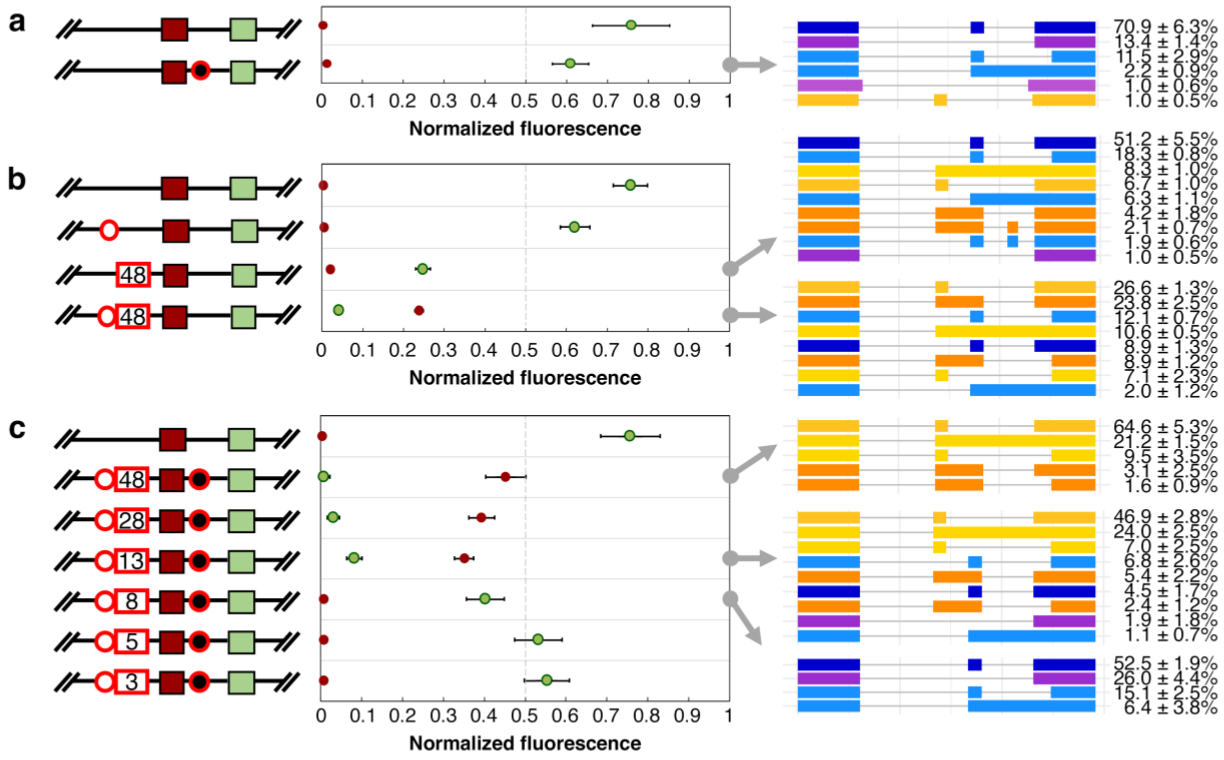

Supplementary Figure 5. Producing alternate isoform profiles with ASD Clover by tuning consensus sequence element strengths. mCherry and Clover fluorescence from ASD Clover devices with a 129-nucleotide exon 2 and a 135-nucleotide exon 3 containing (a) a mutated exon 3 BP element (black circle with red outline), (b) a mutated exon 2 BP element (white circle with red outline) and a mutated exon 2 PPT element (white rectangle with red outline indicating PPT sequence lengths in nucleotides), and (c) serial truncations of modified exon 2 PPT elements with mutations of the BP elements of both exons 2 and 3. Fluorescence from the splicing devices and controls was quantified via flow cytometry. The median fluorescence of each population was measured and normalized to the median fluorescence intensities of the BFP transfection marker and the spliced controls. Median values from biological triplicates were averaged and reported with an error range of ±1 standard deviation. Devices generating distinctive protein isoform profiles were further assessed using long-read sequencing and the relative abundance of each isoform was quantified. Blue represents RNA isoform 1-3-4 and its derivatives; yellow represents RNA isoform 1-2-4 and its derivatives; orange represents RNA isoform 1-2-3-4 and its derivatives; and purple represents RNA isoform 1-4 and its derivatives. The RNA isoform abundance percentages include ±1 standard deviation from biological duplicates. Source data are provided as a Source Data file.

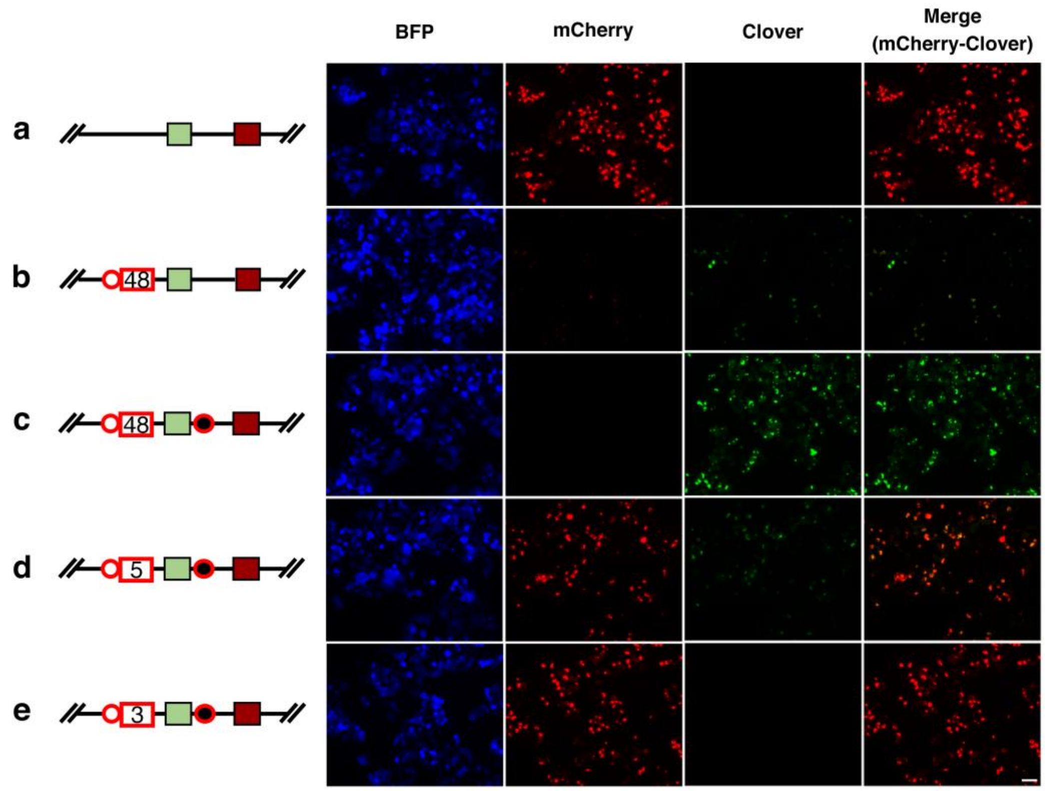

Supplementary Figure 6. Fluorescence microscopy images of ASD mCherry devices in HEK-293T cells. Fluorescence microscopy images of HEK-293T cells transfected with (a) ASD mCherry, (b) an ASD mCherry with a mutated exon 2 BP element (white circle with red outline) and a mutated exon 2 PPT element (white rectangle with red outline indicating PPT sequence lengths in nucleotides), and (c-e) three modified exon 2 PPT elements in ASD mCherry devices with mutations of the BP elements of both exons 2 and 3 (black circle with red outline). BFP is expressed by the transfection marker. mCherry and Clover may be expressed by an ASD. The scale bar represents 50 μm.

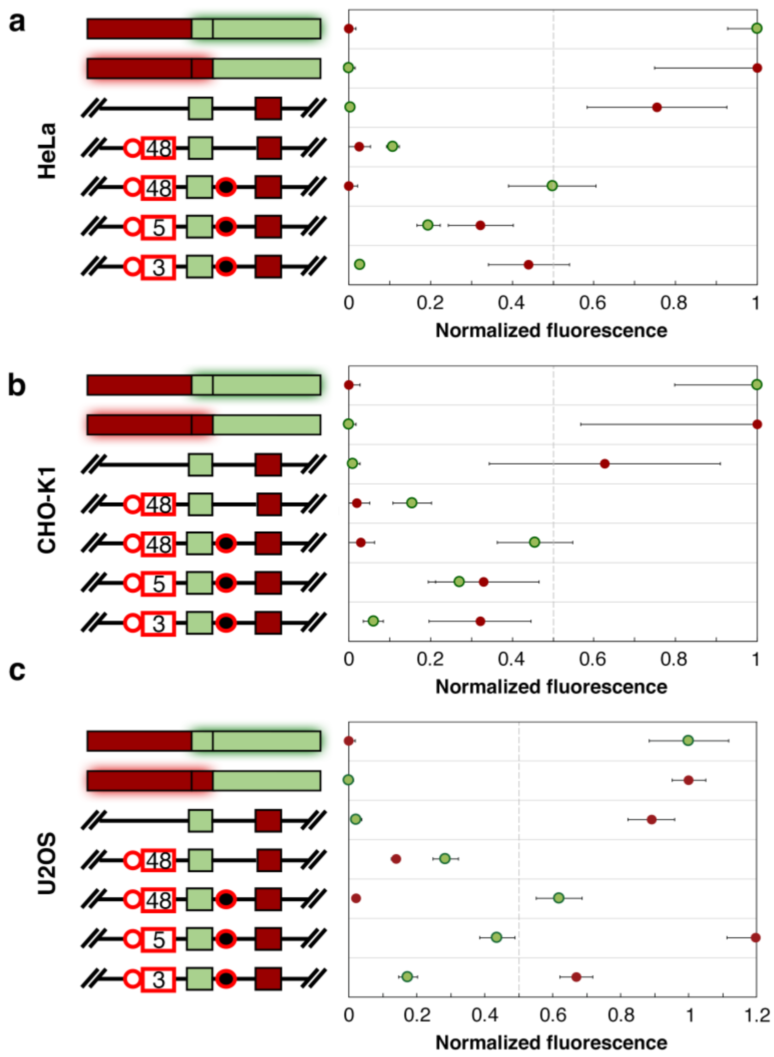

Supplementary Figure 7. Assessment of ASD mCherry devices in HeLa, CHO-K1, and U2OS cells. mCherry and Clover fluorescence from ASD mCherry devices harboring combinations of a mutated exon 3 BP element (black circle with red outline), a mutated exon 2 BP element (white circle with red outline), and truncations of a mutated exon 2 PPT element (white rectangle with red outline indicating PPT sequence lengths in nucleotides) in (a) HeLa, (b) CHO-K1, and (c) U2OS cells. Fluorescence from the splicing devices and controls was quantified via flow cytometry. The median fluorescence of each population was measured and normalized to the median fluorescence intensities of the BFP transfection marker and the spliced controls. Median values from biological triplicates were averaged and reported with an error range of ±1 standard deviation. Source data are provided as a Source Data file.

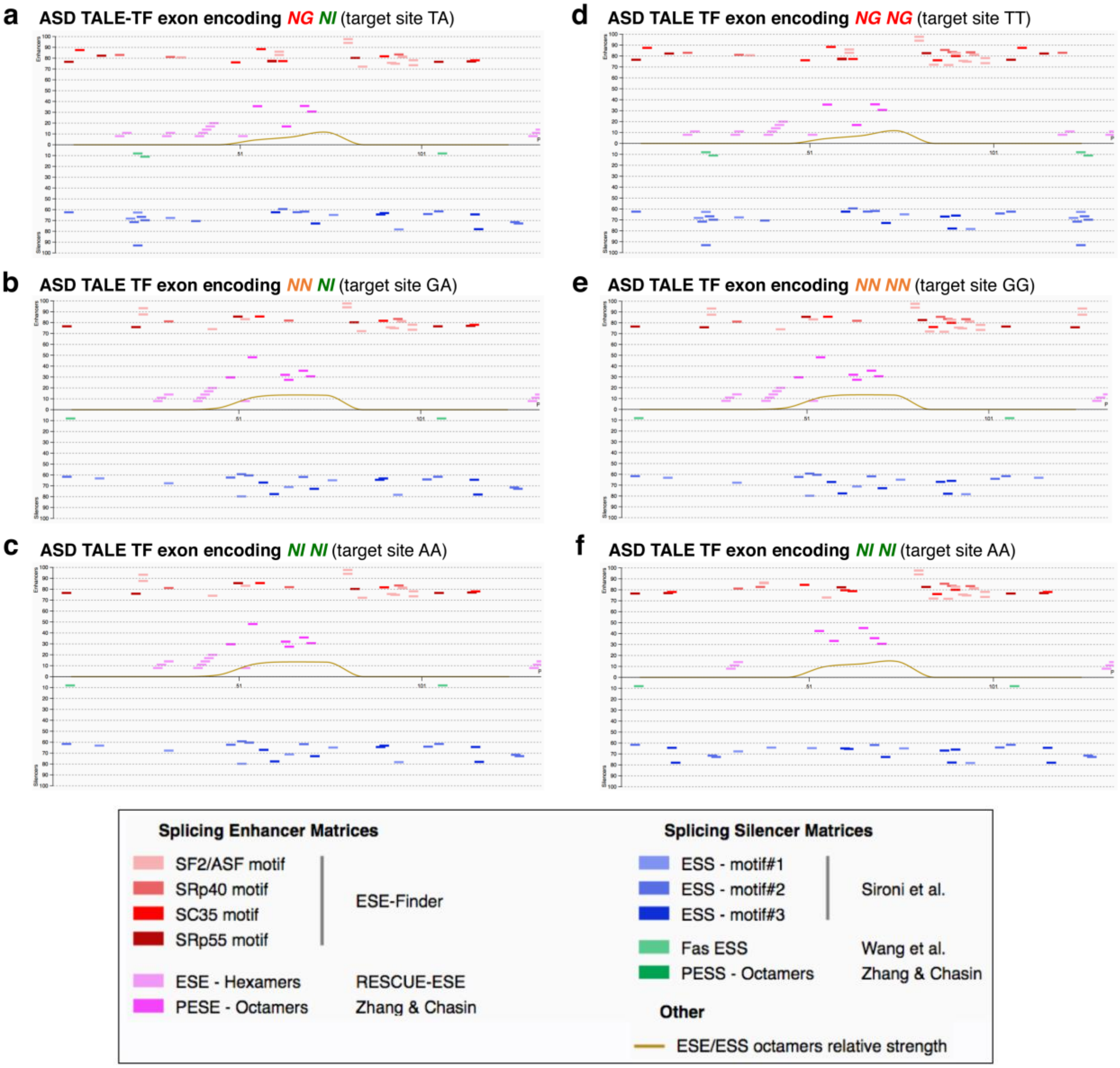

Supplementary Figure 8. Regulatory sequence profiles of mutually exclusive exons in ASD TALE-TFs. The ESE/ESS profiles for an ASD TALE-TF 1 exon encoding RVDs (a) NG NI, (b) NN NI, and (c) NI NI. The ESE/ESS profiles for an ASD TALE-TF 2 exon encoding RVDs (d) NG NG, (e) NN NN, and (f) NI NI. Enhancer motifs are colored in shades of red and pink. Silencer motifs are colored in shades of blue and green. Each color corresponds to motifs from a different prediction algorithm as described in the legend. The yellow line indicates relative ESE/ESS strength.

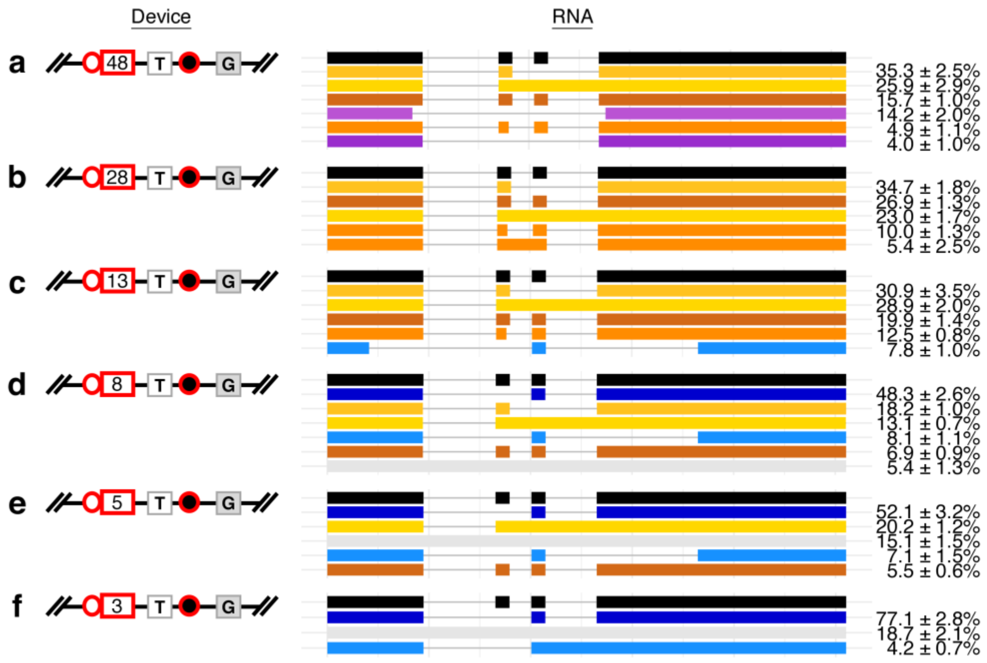

Supplementary Figure 9. RNA isoform profiles from ASD TALE-TF 1 T/G devices. ASD TALE-TF 1 with mutually exclusive exon 2 encoding RVD NG (target site T) and mutually exclusive exon 3 encoding RVD NN (target site G) that contains a mutated exon 3 BP element (black circle with red outline), a mutated exon 2 BP element (white circle with red outline), and serial truncations of exon 2 PPT elements (white rectangle with red outline indicating PPT sequence lengths in nucleotides). Exon 2 PPT elements of (a) 48, (b) 28, (c) 13, (d) 8, (e) 5, and (f) 3 nucleotides were assayed using long-read sequencing and the relative abundance of each isoform was quantified. Blue represents RNA isoform 1-3-4 and its derivatives; yellow represents RNA isoform 1-2-4 and its derivatives; orange represents RNA isoform 1-2-3-4 and its derivatives; purple represents RNA isoform 1-4 and its derivatives; and grey represents unspliced transcript. The RNA isoform abundance percentages include ±1 standard deviation from biological triplicates. Source data are provided as a Source Data file.

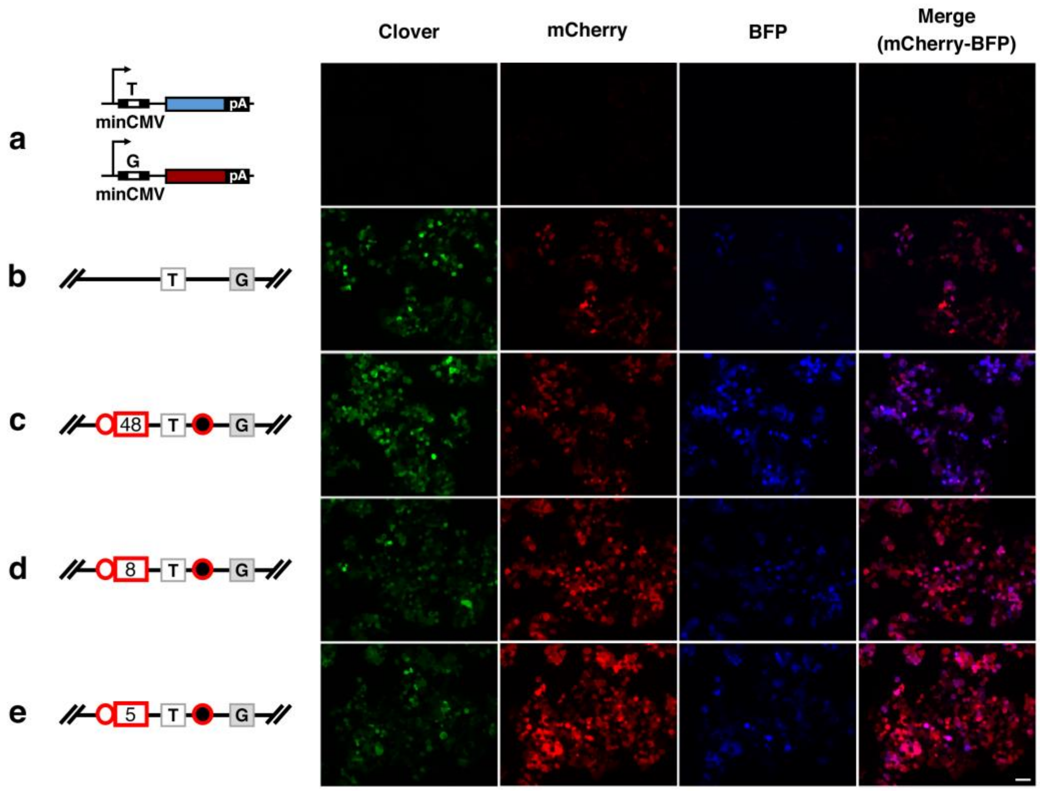

Supplementary Figure 10. Fluorescence microscopy images of ASD TALE-TF 1 T/G devices in HEK-293T cells. Fluorescence microscopy images of HEK-293T cells co-transfected with (a) two binding site reporters, (b) two binding site reporters and ASD TALE-TF 1 T/G, (c-e) two binding site reporters and three truncations of modified exon 2 PPT elements (white rectangle with red outline indicating PPT sequence lengths in nucleotides) in ASD TALE-TF 1 T/G devices with mutations of the BP elements of both exons 2 and 3 (circles with red outlines). Clover is expressed by ASD TALE-TFs. mCherry and BFP expression may be induced by a spliced TALE-TF binding its reporter. The scale bar represents 50 μm.

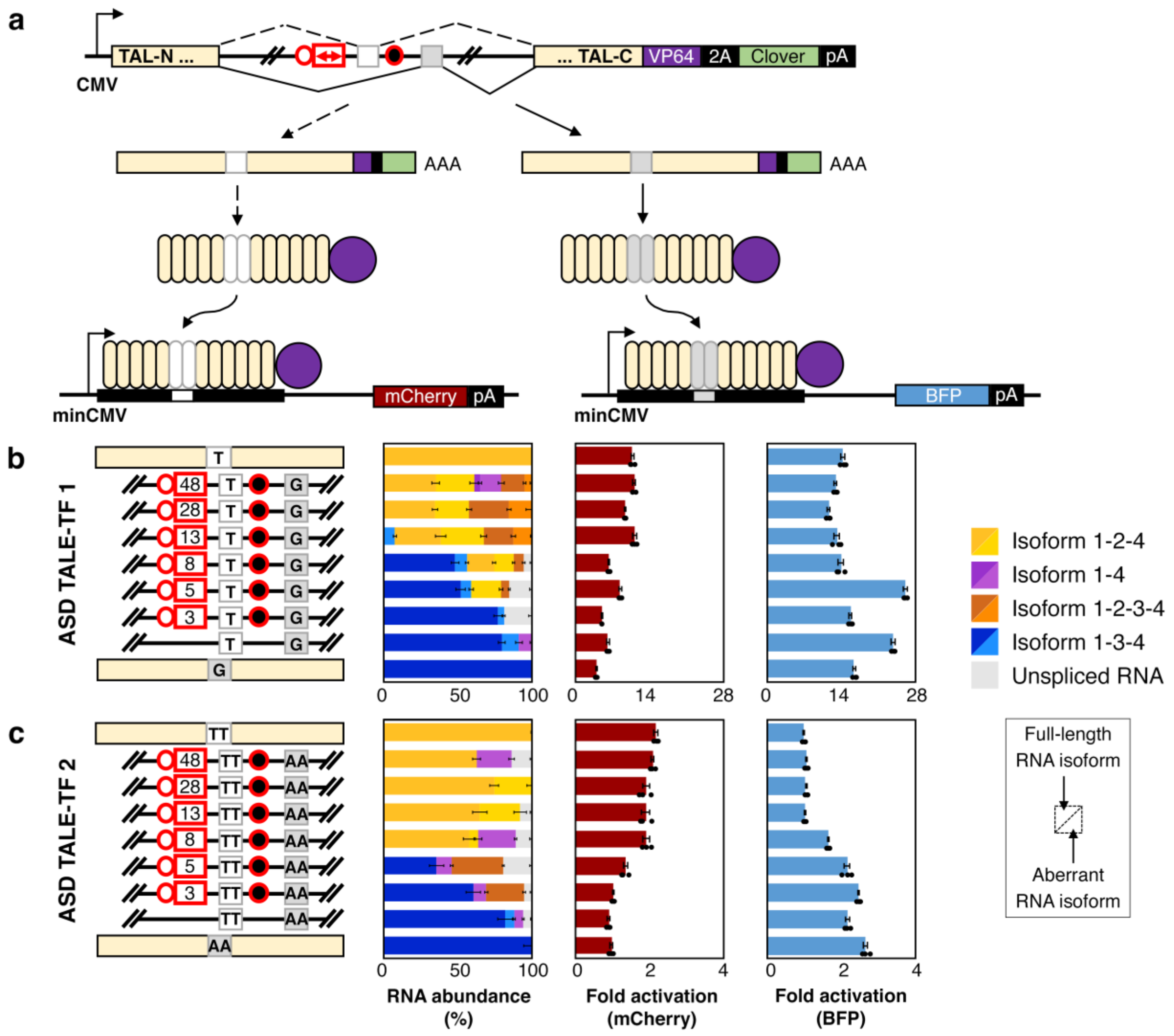

Supplementary Figure 11. Spliced transcription factors activate gene expression from swapped promoter binding sites. (a) Schematic illustrating the activation of two fluorescent proteins from a single mutually exclusive alternative splicing event, where isoform 1-2-4 activates mCherry expression and isoform 1-3-4 activates BFP expression. Devices contain mutated BP elements for exons 2 and 3 (circles with red outlines) and modified exon 2 PPT elements (white rectangle with red outline representing PPT sequence lengths in nucleotides). (b) ASD TALE-TF 1 devices built from a modified intron framework that encodes RVD NG (target site T) in exon 2 and RVD NN (target site G) in exon 3. (c) ASD TALE-TF 2 devices built from a modified intron framework that encodes RVDs NG NG (target site TT) in exon 2 and RVDs NI NI (target site AA) in exon 3. Relative RNA isoform abundances were characterized using long-read sequencing. Error bars in the RNA isoform data represent ±1 standard deviation from biological triplicates. Blue represents RNA isoform 1-3-4 and its derivatives; yellow represents RNA isoform 1-2-4 and its derivatives; orange represents RNA isoform 1-2-3-4 and its derivatives; purple represents RNA isoform 1-4 and its derivatives; and grey represents unspliced transcript. The fold activation was determined via flow cytometry analysis of BFP and mCherry fluorescence in transfected HEK-293T cells, and calculated as the ratio of the median BFP or mCherry fluorescence intensity of cells co-transfected with and without the specified ASD TALE-TF. Fold activations from biological triplicates were averaged and reported within an error range of ±1 standard deviation. Source data are provided as a Source Data file.

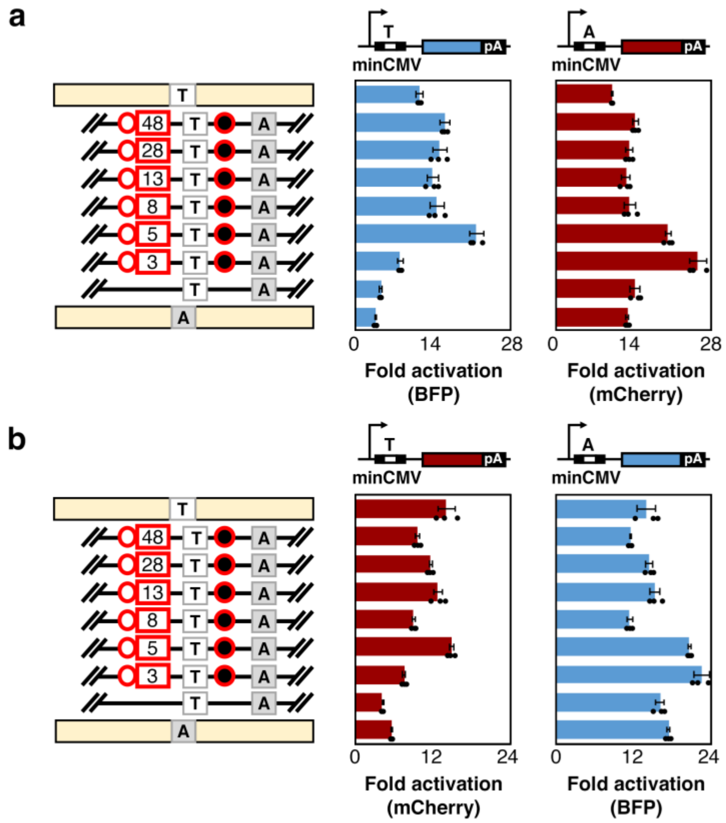

Supplementary Figure 12. Gene activation with ASD TALE-TF 1 T/A from promoter binding sites harboring a one-nucleotide mismatch. ASD TALE-TF 1 devices built from a modified intron framework that encodes RVD NG (target site T) in exon 2 and RVD NI (target site A) in exon 3. (a) Isoform 1-2-4 activates BFP expression and isoform 1-3-4 activates mCherry expression. (b) Isoform 1-2-4 activates mCherry expression and isoform 1-3-4 activates BFP expression. The fold activation was determined via flow cytometry analysis of BFP and mCherry fluorescence in transfected HEK-293T cells, and calculated as the ratio of the median BFP or mCherry fluorescence intensity of cells co-transfected with and without the specified ASD TALE-TF. Fold activations from biological triplicates were averaged and reported within an error range of ±1 standard deviation. Source data are provided as a Source Data file.

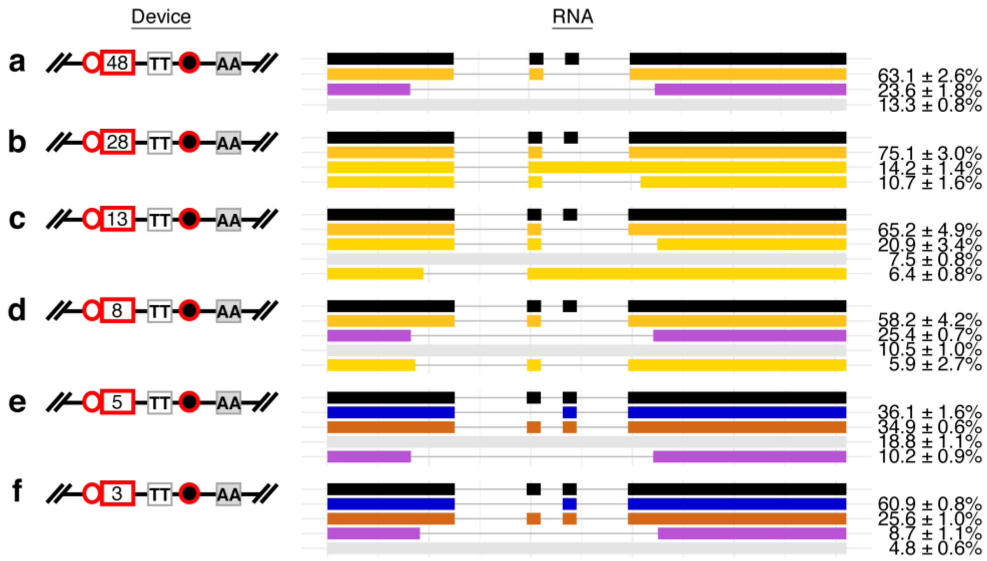

Supplementary Figure 13. RNA isoform profiles for ASD TALE-TF 2 TT/AA devices. ASD TALE-TF 2 with mutually exclusive exon 2 encoding RVDs NG NG (target site TT) and mutually exclusive exon 3 encoding RVDs NI NI (target site AA) that contain a mutated exon 3 BP element (black circle with red outline), a mutated exon 2 BP element (white circle with red outline), and serial truncations of exon 2 PPT elements (white rectangle with red outline indicating PPT sequence lengths in nucleotides). Exon 2 PPT elements of (a) 48, (b) 28, (c) 13, (d) 8, (e) 5, and (f) 3 nucleotides were assayed using long-read sequencing and the relative abundance of each isoform was quantified. Blue represents RNA isoform 1-3-4 and its derivatives; yellow represents RNA isoform 1-2-4 and its derivatives; orange represents RNA isoform 1-2-3-4 and its derivatives; purple represents RNA isoform 1-4 and its derivatives; and grey represents unspliced transcript. The RNA isoform abundance percentages include ±1 standard deviation from biological triplicates. Source data are provided as a Source Data file.

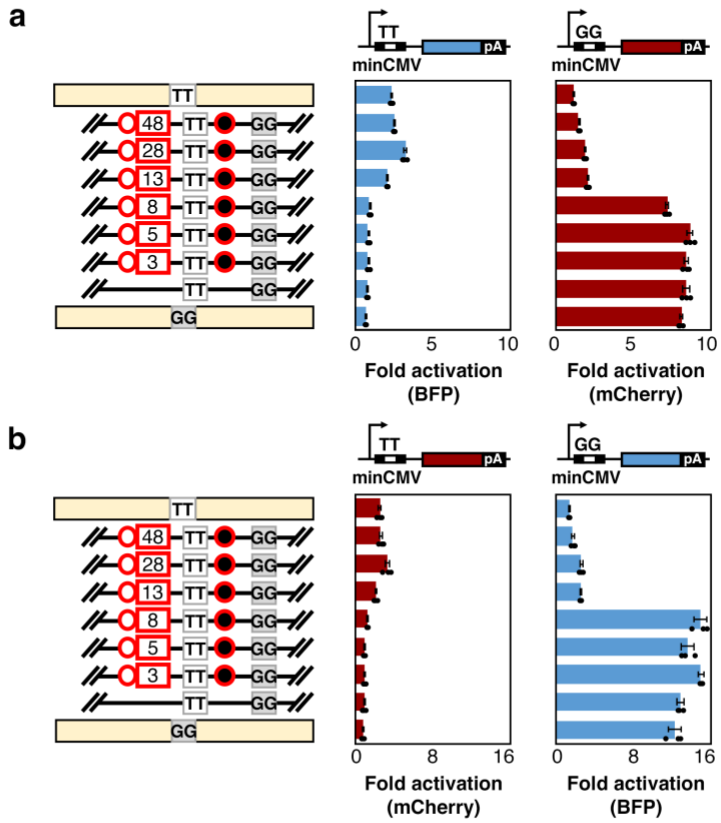

Supplementary Figure 14. Gene activation with ASD TALE-TF 2 TT/GG from promoter binding sites harboring a two-nucleotide mismatch. ASD TALE-TF 2 devices built from a modified intron framework that encodes RVDs NG NG (target site TT) in exon 2 and RVDs NN NN (target site GG) in exon 3. (a) Isoform 1-2-4 activates BFP expression and isoform 1-3-4 activates mCherry expression. (b) Isoform 1-2-4 activates mCherry expression and isoform 1-3-4 activates BFP expression. The fold activation was determined via flow cytometry analysis of BFP and mCherry fluorescence in transfected HEK-293T cells, and calculated as the ratio of the median BFP or mCherry fluorescence intensity of cells co-transfected with and without the specified ASD TALE-TF. Fold activations from biological triplicates were averaged and reported within an error range of ±1 standard deviation. Source data are provided as a Source Data file.

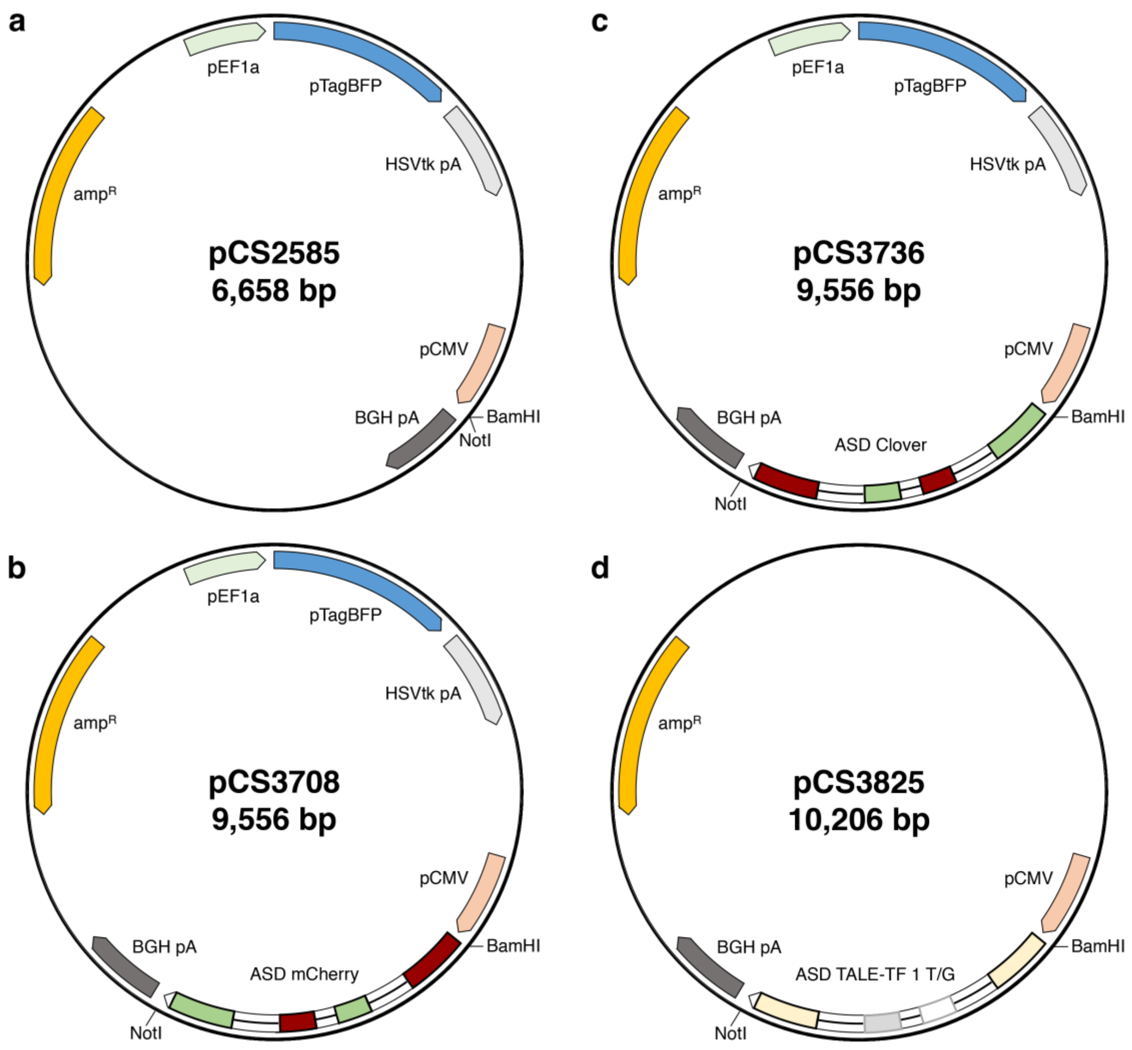

Supplementary Figure 15. Plasmid maps. (a) pCS2585 is the backbone for all of the fluorescent ASDs. It was modified from pCS218 with the integration of pTagBFP in an expression cassette with the EF1a promoter (pEF1a) and HSVtk poly A signal (HSVtk pA) at the BglII restriction site. The plasmid also contains an ampicillin resistance gene (amp^R) and a multi-cloning site between the CMV promoter (pCMV) and BGH poly A signal (BGH pA). (b) pCS3708 encodes ASD mCherry and (c) pCS3736 encodes ASD Clover in pCS2585 between the BamHI and NotI restriction sites. (d) pCS3825 contains ASD TALE-TF 1 T/G in an expression cassette with a CMV promoter and BGH poly A signal between the BamHI and NotI restriction sites in pCS218.

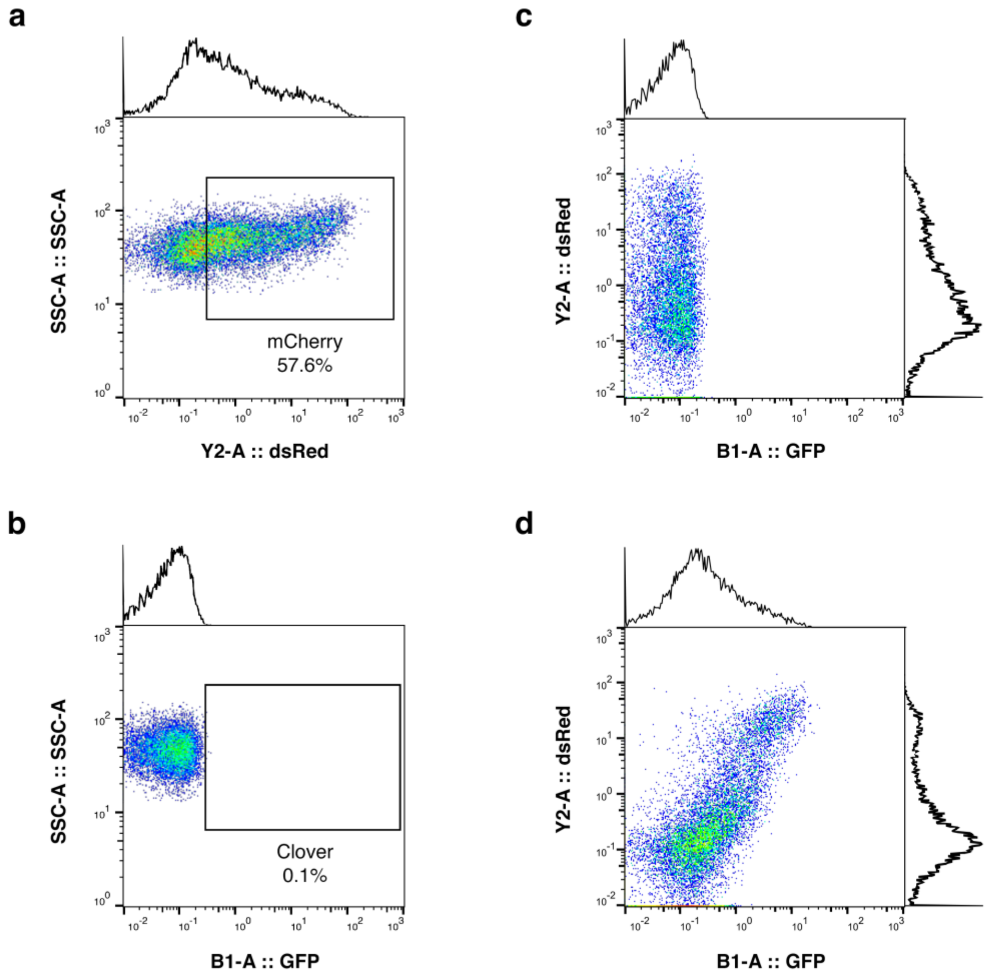

Supplementary Figure 16. Representative flow cytometry plots exemplifying the gating strategy for ASD mCherry. Dot-plots and adjunct histograms exemplifying the gating strategy for ASD mCherry. Viability was gated by SSC-A vs. FSC-A followed by gating for singlets by FSC-H vs. FSC-A. Singlets were further gated for BFP-positive cells, where BFP served as an internal control for transfection efficiency, by SSC-A vs. V1-A. In BFP-positive cells, (a) mCherry-positive cells were gated for by SSC-A vs. Y2-A and (b) Clover-positive cells were gated for by SSC-A vs. B1-A. Cell percentages for the mCherry-positive and Clover-positive populations are noted in the plots. Dot-plots and adjunct histograms for mCherry vs. Clover in BFP-positive cells with (c) ASD mCherry and (d) an ASD mCherry device harboring two BP element mutations and a modified exon 2 PPT element of 5 nucleotides in length.

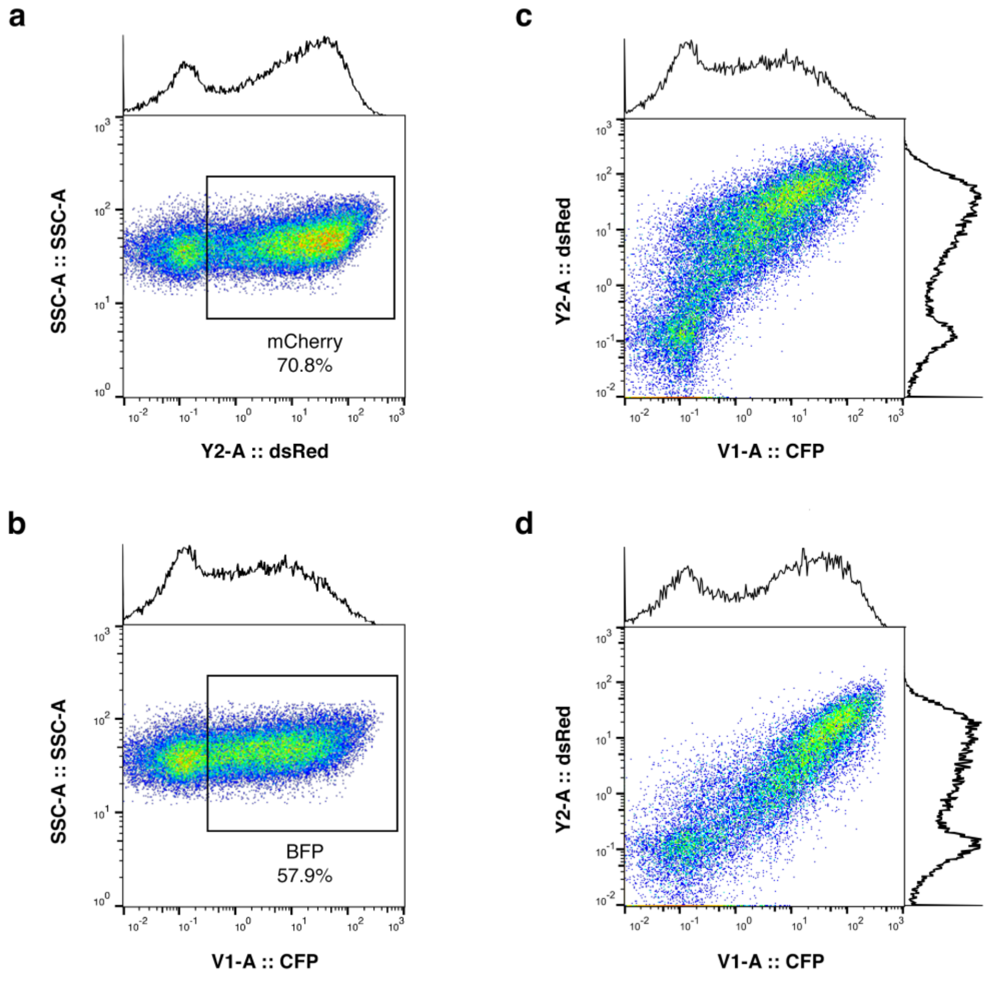

Supplementary Figure 17. Representative flow cytometry plots exemplifying the gating strategy for ASD TALE-TF 1 T/G. Dot-plots and adjunct histograms exemplifying the gating strategy for ASD TALE-TF 1 T/G. Viability was gated by SSC-A vs. FSC-A followed by gating for singlets by FSC-H vs. FSC-A. In singlets, (a) mCherry-positive cells were gated for by SSC-A vs. Y2-A and (b) BFP-positive cells were gated for by SSC-A vs. V1-A. Cell percentages for the mCherry-positive and BFP-positive populations are noted in the plots. Dot-plots and adjunct histograms for mCherry vs. BFP in singlets with (c) ASD TALE-TF 1 T/G and (d) an ASD TALE-TF 1 T/G device harboring two BP element mutations and a modified exon 2 PPT element of 48 nucleotides in length.

Supplementary Table 1. List of plasmids used in this study.

Plasmid Number	Description	Source
pCS218	pcDNA5/FRT	(1)
pCS2585	pCS218-BFP	This work
pCS2586	pCS218-BFP-Clover	This work
pCS2587	pCS218-BFP-mCherry	This work
pCS3703	pCS218-mCherry	This work
pCS3704	pCS218-Clover	This work
pCS3707	TPM1 minigene (TS23D)	(2)
pCS3708	ASD mCherry	This work
pCS3709	ASD mCherry pre-spliced control 1-2-4	This work
pCS3710	ASD mCherry pre-spliced control 1-3-4	This work
pCS3711	ASD mCherry pre-spliced control 1-4	This work
pCS3712	ASD mCherry pre-spliced control 1-2-3-4	This work
pCS3713	ASD mCherry Ex2-144 Ex3-150	This work
pCS3734	ASD mCherry exon 3 branchpoint mutant	This work
pCS3736	ASD Clover	This work
pCS3737	ASD Clover pre-spliced control 1-2-4	This work
pCS3738	ASD Clover pre-spliced control 1-3-4	This work
pCS3739	ASD Clover pre-spliced control 1-4	This work
pCS3740	ASD Clover pre-spliced control 1-2-3-4	This work
pCS3741	ASD Clover Ex2-126 Ex3-132	This work
pCS3762	ASD Clover exon 3 branchpoint mutant	This work
pCS3771	ASD mCherry exon 2 PPT mutant	This work
pCS3772	ASD mCherry exon 2 branchpoint and PPT mutant	This work
pCS3776	ASD mCherry exon 2 branchpoint PPT and exon 3 branchpoint mutant	This work
pCS3777	ASD mCherry exon 2 branchpoint PPT-28 and exon 3 branchpoint mutant	This work
pCS3778	ASD mCherry exon 2 branchpoint PPT-13 and exon 3 branchpoint mutant	This work
pCS3779	ASD mCherry exon 2 branchpoint PPT-8 and exon 3 branchpoint mutant	This work
pCS3780	ASD mCherry exon 2 branchpoint PPT-5 and exon 3 branchpoint mutant	This work
pCS3781	ASD mCherry exon 2 branchpoint PPT-3 and exon 3 branchpoint mutant	This work
pCS3789	ASD Clover exon 2 PPT mutant	This work
pCS3790	ASD Clover exon 2 branchpoint PPT mutant	This work
pCS3793	ASD Clover exon 2 branchpoint PPT and exon 3 branchpoint mutant	This work
pCS3794	ASD Clover exon 2 branchpoint PPT-28 and exon 3 branchpoint mutant	This work

pCS3795	ASD Clover exon 2 branchpoint PPT-13 and exon 3 branchpoint mutant	This work
pCS3796	ASD Clover exon 2 branchpoint PPT-8 and exon 3 branchpoint mutant	This work
pCS3797	ASD Clover exon 2 branchpoint PPT-5 and exon 3 branchpoint mutant	This work
pCS3798	ASD Clover exon 2 branchpoint PPT-3 and exon 3 branchpoint mutant	This work
pCS3809	pLenti-EF1a-Backbone(NI)	(3)
pCS3810	Monomer-NN	(3)
pCS3811	Monomer-NI	(3)
pCS3812	Monomer-NG	(3)
pCS3813	Monomer-HD	(3)
pCS3814	dTALE 1	(3)
pCS3815	dTALE 13	(3)
pCS3816	pAAV-minCMV-mCherry	(3)
pCS3817	TALE 1A	This work
pCS3818	TALE 1T	This work
pCS3819	TALE 1G	This work
pCS3820	TALE 1C	This work
pCS3821	BS 1A-mCherry	This work
pCS3822	BS 1T-mCherry	This work
pCS3823	BS 1G-mCherry	This work
pCS3824	BS 1C-mCherry	This work
pCS3825	ASD TALE 1 T/G	This work
pCS3826	ASD TALE 1 G/T	This work
pCS3827	ASD TALE 1 G/A	This work
pCS3828	ASD TALE 1 A/G	This work
pCS3829	ASD TALE 1 A/T	This work
pCS3830	ASD TALE 1 T/A	This work
pCS3831	TALE 2AA	This work
pCS3832	TALE 2TT	This work
pCS3833	TALE 2GG	This work
pCS3834	TALE 2CC	This work
pCS3835	BS 2AA-mCherry	This work
pCS3836	BS 2TT-mCherry	This work
pCS3837	BS 2GG-mCherry	This work
pCS3838	BS 2CC-mCherry	This work
pCS3839	ASD TALE 2 TT/GG	This work
pCS3840	ASD TALE 2 GG/TT	This work
pCS3841	ASD TALE 2 TT/AA	This work
pCS3842	ASD TALE 2 AA/TT	This work

pCS3843	ASD TALE 2 TT/CC	This work
pCS3844	ASD TALE 2 CC/TT	This work
pCS3845	ASD TALE 1 T/G exon 2BP-PPT3 mutBP	This work
pCS3848	ASD TALE 1 T/A exon 2BP-PPT3 mutBP	This work
pCS3849	ASD TALE 2 TT/GG exon 2BP-PPT3 mutBP	This work
pCS3851	ASD TALE 2 TT/AA exon 2BP-PPT3 mutBP	This work
pCS3853	ASD TALE 1 T/G exon 2BP-PPT3-28 mutBP	This work
pCS3854	ASD TALE 1 T/G exon 2BP-PPT3-13 mutBP	This work
pCS3855	ASD TALE 1 T/G exon 2BP-PPT3-8 mutBP	This work
pCS3856	ASD TALE 1 T/G exon 2BP-PPT3-5 mutBP	This work
pCS3857	ASD TALE 1 T/G exon 2BP-PPT3-3 mutBP	This work
pCS3868	ASD TALE 1 T/A exon 2BP-PPT3-28 mutBP	This work
pCS3869	ASD TALE 1 T/A exon 2BP-PPT3-13 mutBP	This work
pCS3870	ASD TALE 1 T/A exon 2BP-PPT3-8 mutBP	This work
pCS3871	ASD TALE 1 T/A exon 2BP-PPT3-5 mutBP	This work
pCS3872	ASD TALE 1 T/A exon 2BP-PPT3-3 mutBP	This work
pCS3873	ASD TALE 2 TT/GG exon 2BP-PPT3-28 mutBP	This work
pCS3874	ASD TALE 2 TT/GG exon 2BP-PPT3-13 mutBP	This work
pCS3875	ASD TALE 2 TT/GG exon 2BP-PPT3-8 mutBP	This work
pCS3876	ASD TALE 2 TT/GG exon 2BP-PPT3-5 mutBP	This work
pCS3877	ASD TALE 2 TT/GG exon 2BP-PPT3-3 mutBP	This work
pCS3883	ASD TALE 2 TT/AA exon 2BP-PPT3-28 mutBP	This work
pCS3884	ASD TALE 2 TT/AA exon 2BP-PPT3-13 mutBP	This work
pCS3885	ASD TALE 2 TT/AA exon 2BP-PPT3-8 mutBP	This work
pCS3886	ASD TALE 2 TT/AA exon 2BP-PPT3-5 mutBP	This work
pCS3887	ASD TALE 2 TT/AA exon 2BP-PPT3-3 mutBP	This work
pCS3893	BS 1A-BFP	This work
pCS3894	BS 1T-BFP	This work
pCS3895	BS 1G-BFP	This work
pCS3896	BS 1C-BFP	This work
pCS3897	BS 2AA-BFP	This work
pCS3898	BS 2TT-BFP	This work
pCS3899	BS 2GG-BFP	This work
pCS3900	BS 2CC-BFP	This work

Supplementary Table 2. List of representative primer sequences used in this study.

Name	Sequence (5' → 3')
**pCS2585**	
EF1a F	ATCGGGAGATCTGGATCT
EF1a R	ATATACTCGAGGGTCACAGCTTGGATCT
pTagBFP F	CTCGAGTATTCGCCACCATGAGCGAGCTGATTAAGG
pTagBFP R	GATCGTCTCATGCAGATCAATTAAGCTTGTGCCCCAG
HSVtk F	TGATCTGCATGAGACGATCTCATGCTGGAGTTCTTCG
HSVtk R	ATATACAATTGATGAGTAACCTGAGGCTATG
**ASD mCherry and ASD Clover**	
ASD mCherry_mCherry1 F	TATAGAACCCGAACGACCGAGCGCAGCGGCCGCCACCATGGTGAGCAAGGGCGA
ASD mCherry_mCherry1 R	GTAGACGCAGACCTGCACGGGCTTCTTGGCCTTGTAGGT
ASD mCherry_Intron1 F	GCCAAGAAGCCCGTGCAGGTCTGCGTCTACCCCCAACCC
ASD mCherry_Intron1 R	CTCGCCCTTGCTCACGAGCTGGGACAGCGGAGAGCGGGC
ASD mCherry_Clover1 F	CCGCTGTCCCAGCTCGTGAGCAAGGGCGAGGAGCTGTTC
ASD mCherry_Clover1 R	GCGCCCGGGTACCTTGCCGTTGGTGGCATCGCCCTCGCC
ASD mCherry_Intron2 F	GATGCCACCAACGGCAAGGTACCCGGGCGCGCGGTGTGG
ASD mCherry_Intron2 R	GTTGTAGGCTCCGGGCAGCTGGGAGTTGGCAGTGGAGTA
ASD mCherry_mCherry2 F	GCCAACTCCCAGCTGCCCGGAGCCTACAACGTCAACATC
ASD mCherry_mCherry2 R	GCGTGCACTTACATCCTTGTACAGCTCGTCCATGCCGCC
ASD mCherry_Intron3 F	GACGAGCTGTACAAGGATGTAAGTGCACGCTCACACTGC
ASD mCherry_Intron3 R	GATGAACTTCAGGGTAGCCTAGGCAGAGGATGAAAGACA
ASD mCherry_Clover2 F	TCCTCTGCCTAGGCTACCCTGAAGTTCATCTGCACCACC
ASD mCherry_Clover2 R	AGTTACGCGCTTCCTCGCTCACTGACTTTATTATGATCAGTTACTTGTACAGCT
ASD Clover_Clover1 F	TATAGAACCCGAACGACCGAGCGCAGCGGCCGCCACCATGGTGAGCAAGGGCGA
ASD Clover_Clover1 R	GGGGTAGACGCAGACCTGGTGGTTGTCGGGCAGCAGCAC

ASD Clover_Intron1 F	CCCGACAACCACCAGGTCTGCGTCTACCCCCAAC CCCCA
ASD Clover_Intron1 R	GCCGGAGGAGCCGAGCTGGGACAGCGGAGAGC GGGCGCG
ASD Clover_mCherry1 F	TCTCCGCTGTCCCAGCTCGGCTCCTCCGGCTCCT CCGTG
ASD Clover_mCherry1 R	CGCGCGCCCGGGTACCTTGCCCTCGATCTCGAAC TCGTG
ASD Clover_Intron2 F	GAGATCGAGGGCAAGGTACCCGGGCGCGCGGTG TGGCAC
ASD Clover_Intron2 R	GGACTGATGGCTCAGCTGGGAGTTGGCAGTGGA GTACGG
ASD Clover_Clover2 F	ACTGCCAACTCCCAGCTGAGCCATCAGTCCGCCC TGAGC
ASD Clover_Clover2 R	TGAGCGTGCACTTACATCGGAGGAGCCGGAGGA GCCCTT
ASD Clover_Intron3 F	TCCGGCTCCTCCGATGTAAGTGCACGCTCACACT GCCTC
ASD Clover_Intron3 R	GGGGCGGCCCTCGCCCTAGGCAGAGGATGAAAG ACACTC
ASD Clover_mCherry2 F	TCATCCTCTGCCTAGGGCGAGGGCCGCCCCTACG AGGGC
ASD Clover_mCherry2 R	AGTTACGCGCTTCCTCGCTCACTGACTTTATTAT GATCAGTTACTTGTACAGCT
pCS2585 BamHI F	TTAAGCTTGGTACCGAGCTC
pCS2585 NotI R	CTGATCAGCGGGTTTAAACG
**Reverse transcription**	
BGH R	TAGAAGGCACAGTCGAGG
**Intronic modifications**	
ASD mCherry exon 3 mutBP F	ACGAATGGCGATCTTTCTCTTTCTCTCTCCCTC
ASD mCherry exon 3 mutBP R	GAAAGATCGCCATTCGTGTGCAGTGCC
ASD mCherry 2BP3 F	TCCCTTAGCCCCAGGACCCCGGCTAACCTGGCAC CCGT
ASD mCherry 2BP3 R	GGGGTCCTGGGGCTAAGGGATCGCTGCTTTG
ASD mCherry 2BPPPT3 F	CTTAGCCCCAGGACCCCGGCTAACTTTCTCTTTC TCTCTC
ASD mCherry 2BPPPT3 R	GAGCCGGAGGAGCCGAGCTGGGAAAGAGAGAG AGAGGGA
ASD mCherry-2BPPPT3-truncations_28nt F	TCTCTCTCCCTCCCTGTCTTAGCTCGGCTC
ASD mCherry-2BPPPT3-truncations_13nt F	GGCTAACTTTCTCTTTCTCTAGCTCGGCTC
ASD mCherry-2BPPPT3-truncations_8nt F	ACCCCGGCTAACTTTCTCTTAGCTCGGCTC
ASD mCherry-2BPPPT3-truncations_5nt F	AGGACCCCGGCTAACTTTCTAGCTCGTGAG

ASD mCherry-2BPPPT3-truncations_3nt F	CCAGGACCCCGGCTAACTTTAGCTCGTGAG
ASD mCherry-2BPPPT3-truncations_3nt R	AAAGTTAGCCGGGGTCCTGGGGCTAAGGGA
**TALE monomers**	
TAL F1	ATATAGATGCCGTCCTAGCGCGTCTCCTGACCCC AGAGCAGGTCGTGG
TAL F2	TGCTCTTTATTCGTTGCGTCGGTCTCGACTCACCC CAGAGCAGGTCGTG
TAL F3	TGCTCTTTATTCGTTGCGTCGGTCTCGCCTCACCC CAGAGCAGGTCGTG
TAL F4	TGCTCTTTATTCGTTGCGTCGGTCTCGATTAACCC CAGAGCAGGTCGTG
TAL F5	ATATAGATGCCGTCCTAGCGCGTCTCGCTTAACC CCAGAGCAGGTCGTG
TAL F6	TGCTCTTTATTCGTTGCGTCGGTCTCGACTCACCC CAGAGCAGGTCGTG
TAL F7	TGCTCTTTATTCGTTGCGTCGGTCTCGCCTCACCC CAGAGCAGGTCGTG
TAL F8	TGCTCTTTATTCGTTGCGTCGGTCTCGATTAACCC CAGAGCAGGTCGTG
TAL F9	ATATAGATGCCGTCCTAGCGCGTCTCGGCTCACC CCAGAGCAGGTCGTG
TAL F10	TGCTCTTTATTCGTTGCGTCGGTCTCGACTCACCC CAGAGCAGGTCGTG
TAL F11	TGCTCTTTATTCGTTGCGTCGGTCTCGCCTCACCC CAGAGCAGGTCGTG
TAL F12	TGCTCTTTATTCGTTGCGTCGGTCTCGATTAACCC CAGAGCAGGTCGTG
TAL R1	TCTTATCGGTGCTTCGTTCTGGTCTCTGAGTCCGT GCGCTTGGCAC
TAL R2	TCTTATCGGTGCTTCGTTCTGGTCTCTGAGGCCG TGCGCTTGGCAC
TAL R3	TCTTATCGGTGCTTCGTTCTGGTCTCTTAATCCGT GCGCTTGGCAC
TAL R4	AAGTATCTTTCCTGTGCCCACGTCTCTTAAGCCG TGCGCTTGGCAC
TAL R5	TCTTATCGGTGCTTCGTTCTGGTCTCTGAGTCCGT GCGCTTGGCAC
TAL R6	TCTTATCGGTGCTTCGTTCTGGTCTCTGAGGCCG TGCGCTTGGCAC
TAL R7	TCTTATCGGTGCTTCGTTCTGGTCTCTTAATCCGT GCGCTTGGCAC
TAL R8	AAGTATCTTTCCTGTGCCCACGTCTCTGAGCCCG TGCGCTTGGCAC
TAL R9	TCTTATCGGTGCTTCGTTCTGGTCTCTGAGTCCGT GCGCTTGGCAC

TAL R10	TCTTATCGGTGCTTCGTTCTGGTCTCTGAGGCCG TGCGCTTGGCAC
TAL R11	TCTTATCGGTGCTTCGTTCTGGTCTCTTAATCCGT GCGCTTGGCAC
TAL R12	AAGTATCTTTCCTGTGCCCACGTCTCTGAGTCCG TGCGCTTGGCAC
TAL F-assem	ATATAGATGCCGTCCTAGCG
TAL R-assem	AAGTATCTTTCCTGTGCCCA
**ASD TALE-TF devices**	
TALE F6 BsaI-0.5NG Int1A F	CAACGGTCTCGACTCACCCCAGAGCAGGTCTGC GTCTACCCCCAA
TALE R8 BsmBI Int1A R	CAACCGTCTCTTTAGGACTTAATCCTTGCCCCG
TALE F9 BsmBI Int1B F	CAACCGTCTCCCTAAGGTGTCCGAAGTTCTTTGG
TALE R12 BsmBI Int1B filler R	CAACCGTCTCTGAGTCACCACCTGGGACAGCGG AGAG
TALE Int1 R	CTGGGACAGCGGAGAG
TALE NG 7 Int3B F	CTTTCATCCTCTGCCTAGGCGTTGGAAACCGTAC A
TALE F1 BsmBI Int3A filler F	CAACCGTCTCCTGACCACCACGTAAGTGCACGCT CACA
TALE Int3 F	GTAAGTGCACGCTCACA
TALE R4 BsmBI Int3A R	CAACCGTCTCTCAGCAGTCCCCATTTCAGAGGC
TALE F5 BsmBI Int3B F	CAACCGTCTCGGCTGGAAGTGAACTTCGCCTC
TALE F1 VVAIAS NG Int 1 BsmBI F	CAACCGTCTCCTGACCGCTCTCCGCTGTCCCAGG TCGTGGCCATTGCCT
TALE R4 NGGGKQ NG Int 2 R	CAACCGTCTCTGTACCTGTTTGCCCCCTCCATT
TALE F5 Int2 BsmBI F	CAACCGTCTCGGTACCCGGGCGCGCGGTGT
TALE R8 Int2 BsmBI R	CAACCGTCTCTCTGGGAGTTGGCAGTGGAGTA
TALE F9 VVAIAS HD Int2 BsmBI F	CAACCGTCTCGCCAGGTCGTGGCGATCGCAAGC
TALE R12 HDGGKQ Int3 R	CAACCGTCTCTGAGTCAGTGTGAGCGTGCACTTA CTTGCTTTCCTCCGTCGTG
TALE F1 VVAIAS HD Int1 BsmBI F	CAACCGTCTCCTGACCGCTCTCCGCTGTCCCAGG TCGTGGCGATCGCAAGC
TALE R4 HDGGKQ Int 2 R	CAACCGTCTCTGTACTTGCTTTCCTCCGTCGTG
TALE F9 VVAIAS NG Int2 BsmBI F	CAACCGTCTCGCCAGGTCGTGGCCATTGCCT
TALE R12 NGGGKQ Int3 R	CAACCGTCTCTGAGTCAGTGTGAGCGTGCACTTA CCTGTTTGCCCCCTCCATT
TALE Int1 F Part 3	CGCTCTCCGCTGTCCCAG
TALE Int3 R Part 3	CAGTGTGAGCGTGCACTTAC
TALE Int3B R Stitch	CTAGGCAGAGGATGAAAG
TALE Int1 F Part 2 BsmBI	CAACCGTCTCCTGACCGCTCTCCGCTGTCCCAG
TALE Int3 R Part 2 BsmBI	CAACCGTCTCTGTACCTG
TALE Int2 R BsmBI bind	CAACCGTCTCGCCAGGTC

TALE Int2 F BsmBI bind	CAACCGTCTCTGAGTCAGTGTGAGCGTGCACTTAC
TALE F1 VVAIAS NI Int1 BsmBI F	CAACCGTCTCCTGACCGCTCTCCGCTGTCCCAGGTCGTGGCAATCGCCTCC
TALE R4 NIGGKQ Int 2 R	CAACCGTCTCTGTACCTGTTTCCCGCCAATGTT
TALE F9 VVAIAS NI Int2 BsmBI F	CAACCGTCTCGCCAGGTCGTGGCAATCGCCTCC
TALE R12 NIGGKQ Int3 R	CAACCGTCTCTGAGTCAGTGTGAGCGTGCACTTACCTGTTTCCCGCCAATGTT
TALE F1 VVAIAS NN Int1 BsmBI F	CAACCGTCTCCTGACCGCTCTCCGCTGTCCCAGGTCGTGGCAATCGCGAGC
TALE R4 NNGGKQ Int2 R	CAACCGTCTCTGTACCTGTTTTCCGCCGTTATT
TALE F9 VVAIAS NN Int2 BsmBI F	CAACCGTCTCGCCAGGTCGTGGCAATCGCGAGC
TALE R12 NNGGKQ Int3 R	CAACCGTCTCTGAGTCAGTGTGAGCGTGCACTTACCTGTTTTCCGCCGTTATT
pCS218 ASD F	AAGCTTGGTACCGAGCTCGGATCCCGCCACCATGTCGCGGAC
pCS218 ASD R	CCTCTAGACTCGAGCGGCCGCTTATGATCAGTTACTTGTACAGCTCGTCC
Ex1verif F	CACGGGAGCACCCCTCAAC
Ex4verif R	ACTCCAGTGCGGGTCTGC
**TALE reporters**	
TALE 1A BS F	CTAGATAATACAACTCACTATAGGGG
TALE 1A BS R	GATCCCCCTATAGTGAGTTGTATTAT
TALE 1T BS F	CTAGATAATACTACTCACTATAGGGG
TALE 1T BS R	GATCCCCCTATAGTGAGTAGTATTAT
TALE 1G BS F	CTAGATAATACGACTCACTATAGGGG
TALE 1G BS R	GATCCCCCTATAGTGAGTCGTATTAT
TALE 1C BS F	CTAGATAATACCACTCACTATAGGGG
TALE 1C BS R	GATCCCCCTATAGTGAGTGGTATTAT
TALE 2AA BS F	CTAGATAATTTTGTAATGTTTAGGGG
TALE 2AA BS R	GATCCCCCTAAACATTACAAAATTAT
TALE 2TT BS F	CTAGATAATTTTGTTTTCTTTAGGGG
TALE 2TT BS R	GATCCCCCTAAAGAAAACAAAATTAT
TALE 2GG BS F	CTAGATAATTTTGTGGTCTTTAGGGG
TALE 2GG BS R	GATCCCCCTAAAGACCACAAAATTAT
TALE 2CC BS F	CTAGATAATTTTGTCCTCTTTAGGGG
TALE 2CC BS R	GATCCCCCTAAAGAGGACAAAATTAT

Supplementary Table 3. List of intron sequences used in this study.

Description	Sequence (5’ → 3’)	Source
Intron Framework Intron 1	GTCTGCGTCTACCCCCAACCCCCACTGCCCCAACTCC<br>GCGTCAGTCTCTCAGTCCGGGAACCCGAACTGGCTCC<br>CTTTCCCCATCCTATCCCCAGCACAGGATGTCCAGGG<br>GCGCGCATTTAAAAAAAAAATTAGGAGAAGAAGCTC<br>GAGAGTTAGACTGATTCTAACTTTTCTGTCTCGCCTG<br>AGGACGCTTGTGGCCCTTCTCTGGCTCCCACGGTCCG<br>CGAAGTCCACTGGAATTCCCCGTAAAGAGGCTCTTG<br>ACTCTTAGGATTGGGGATCAGTTTTGAAAGAGGAAG<br>GGAGAAAGGATACAAGTTCCCGGGATGCCTGGAGGG<br>AAGGGCGGGGGAGCGGGGCAAGGATTAAGTCCTAA<br>GGTGTCCGAAGTTCTTTGGACCTTTTGGTTGGGGAGA<br>GAGAGCCCTTAAGATGGGATTGAAAACTTTGAAGCG<br>CACTGAATTATTTCCTAAGAACAAAGATGGGACAGA<br>ATGGGAGACACAGACGGAAAGCTAGCCAAACAAGG<br>GCGTGTTTCTTCAATCCCTCAAGTTGGGGACCTGAGC<br>TTGCTGTCTTCGGCCGAGCGTGTGGTCCCGGGGTGGC<br>GTGGGGTGGGGGGTGGGATGCGAGATGAGTCACTGA<br>GTGGTTCCAGCTCTGGGAGAGAGATTACCTAGGGTC<br>CCTTGAGCCCCAAAGCAGCGATCCCTTAGCCCCAGG<br>ACCCCAGCCAACCTGGCACCCGTTTGTTGTGTGTCTC<br>ACACCCGGTCCATGCCGGCCGCCCGCGCCCGCTCTCC<br>GCTGTCCCAG	(2)
Intron Framework Intron 2	GTACCCGGGCGCGCGGTGTGGCACTGCACACGAATG<br>GCTAACTTTCTCTTTCTCTCTCCCTCCCTGTCTTTCCC<br>TCTCTCTCTCTTTCCCGCTGTCCCTGTCCTTTATGGTC<br>TACGCACCCTCAACCCGCACCTTGCGGGATCACGCTG<br>CCTGCTGCACCCCACCCCCTTCCCCCTTCCTTCCCCCC<br>ACCCCCGTACTCCACTGCCAACTCCCAG	(2)
Intron Framework Intron 3	GTAAGTGCACGCTCACACTGCCTCCCTCACCCCCTGA<br>CCGCGTGGCCGCTCTGGGGGTCACCACAGGGGCTGC<br>AGAGCAAAGGAAGAGGGTGATCCTCCTCCTACAGGA<br>CACCTGCACACAGCCTGGCCATAGCCCAGAGCACTG<br>GATGCCGCCTCTGCTGCTGCGCACATTTCATTTATAT<br>TCTGTCCTTTCCCCTTTTTCTCCTCTTCTTTACCTCCTC<br>CCCTTTGGTTGGAGGTGGGTGGGTGAGAAGCTGGGG<br>AACACGGCCTCTGAAATGGGGACTGCTGGAAGTGAA<br>CTTCGCCTCCTGCTGGTATAAAACCGCTGAAGTGTAT<br>GTCATCACCAAGGTCTGTACAAAACAGAATCCCTAG<br>TGTTCTTGTTTGCCACCCTACCCCCAAAACCCCCGGT<br>GGTTTTCGCTGATGGACCTAGTCTGAGTGGGTTCAGA<br>GAGCCTGACCTTTGGAATTCCTCACTTTCTCCCCATC<br>TCTGAGTGTCTTTCATCCTCTGCCTAG	(2)

Supplementary Table 4. List of exon sequences used in this study.
Underlined sequences in the TALE exons correspond to the RVDs for each TALE monomer.

Description	Sequence (5' → 3')	Source
ASD mCherry<br>Exon 1	ATGGTGAGCAAGGGCGAGGAGGATAACATGGCCA<br>TCATCAAGGAGTTCATGCGCTTCAAAGTGCACATG<br>GAGGGCTCCGTGAACGGCCACGAGTTCGAGATCGA<br>GGGCGAGGGCGAGGGCCGCCCCTACGAGGGCACC<br>CAGACCGCCAAGCTGAAAGTGACCAAGGGTGGCCC<br>CCTGCCCTTCGCCTGGGACATCCTGTCCCCTCAGTT<br>CATGTACGGCTCCAAGGCCTACGTGAAGCACCCCG<br>CCGACATCCCCGACTACTTGAAGCTGTCCTTCCCCG<br>AGGGCTTCAAGTGGGAGCGCGTGATGAACTTCGAG<br>GACGGCGGCGTGGTGACCGTGACCCAGGACTCCTC<br>CCTGCAGGACGGCGAGTTCATCTACAAAGTGAAGC<br>TGCGCGGCACCAACTTCCCCTCCGACGGCCCCGTA<br>ATGCAGAAGAAGACCATGGGCTGGGAGGCCTCCTC<br>CGAGCGGATGTACCCCGAGGACGGCGCCCTGAAGG<br>GCGAGATCAAGCAGAGGCTGAAGCTGAAGGACGG<br>CGGCCACTACGACGCTGAAGTCAAGACCACCTACA<br>AGGCCAAGAAGCCCGTGCAG	pCS3703
ASD mCherry<br>Exon 2	CTCGTGAGCAAGGGCGAGGAGCTGTTCACCGGGGT<br>GGTGCCCATCCTGGTCGAGCTGGACGGCGACGTAA<br>ACGGCCACAAGTTCAGCGTCCGCGGCGAGGGCGAG<br>GGCGATGCCACCAACGGCAAG	pCS3704
ASD mCherry<br>Exon 3	CTGCCCGGAGCCTACAACGTCAACATCAAGTTGGA<br>CATCACCTCCCACAACGAGGACTACACCATCGTGG<br>AACAGTACGAACGCGCCGAGGGCCGCCACTCCACC<br>GGCGGCATGGACGAGCTGTACAAGGAT	pCS3703
ASD mCherry<br>Exon 4	GCTACCCTGAAGTTCATCTGCACCACCGGCAAGCT<br>GCCCGTGCCCTGGCCCACCCTCGTGACCACCTTCGG<br>CTACGGCGTGGCCTGCTTCAGCCGCTACCCCGACC<br>ACATGAAGCAGCACGACTTCTTCAAGTCCGCCATG<br>CCCGAAGGCTACGTCCAGGAGCGCACCATCTCTTT<br>CAAGGACGACGGTACCTACAAGACCCGCGCCGAG<br>GTGAAGTTCGAGGGCGACACCCTGGTGAACCGCAT<br>CGAGCTGAAGGGCATCGACTTCAAGGAGGACGGC<br>AACATCCTGGGGCACAAGCTGGAGTACAACTTCAA<br>CAGCCACAACGTCTATATCACGGCCGACAAGCAGA<br>AGAACGGCATCAAGGCTAACTTCAAGATCCGCCAC<br>AACGTTGAGGACGGCAGCGTGCAGCTCGCCGACCA<br>CTACCAGCAGAACACCCCCATCGGCGACGGCCCCG<br>TGCTGCTGCCCGACAACCACTACCTGAGCCATCAG<br>TCCGCCCTGAGCAAAGACCCCAACGAGAAGCGCGA<br>TCACATGGTCCTGCTGGAGTTCGTGACCGCCGCCG<br>GGATTACACATGGCATGGACGAGCTGTACAAGTAA	pCS3704
ASD Clover<br>Exon 1	ATGGTGAGCAAGGGCGAGGAGCTGTTCACCGGGGT<br>GGTGCCCATCCTGGTCGAGCTGGACGGCGACGTAA<br>ACGGCCACAAGTTCAGCGTCCGCGGCGAGGGCGAG	pCS3704

	GGCGATGCCACCAACGGCAAGCTGACCCTGAAGTT<br>CATCTGCACCACCGGCAAGCTGCCCGTGCCCTGGC<br>CCACCCTCGTGACCACCTTCGGCTACGGCGTGGCCT<br>GCTTCAGCCGCTACCCCGACCACATGAAGCAGCAC<br>GACTTCTTCAAGTCCGCCATGCCCGAAGGCTACGT<br>CCAGGAGCGCACCATCTCTTTCAAGGACGACGGTA<br>CCTACAAGACCCGCGCCGAGGTGAAGTTCGAGGGC<br>GACACCCTGGTGAACCGCATCGAGCTGAAGGGCAT<br>CGACTTCAAGGAGGACGGCAACATCCTGGGGCACA<br>AGCTGGAGTACAACTTCAACAGCCACAACGTCTAT<br>ATCACGGCCGACAAGCAGAAGAACGGCATCAAGG<br>CTAACTTCAAGATCCGCCACAACGTTGAGGACGGC<br>AGCGTGCAGCTCGCCGACCACTACCAGCAGAACAC<br>CCCCATCGGCGACGGCCCCGTGCTGCTGCCCGACA<br>ACCACCAG	
ASD Clover Exon 2	CTCGTGAGCAAGGGCGAGGAGGATAACATGGCCAT<br>CATCAAGGAGTTCATGCGCTTCAAAGTGCACATGG<br>AGGGCTCCGTGAACGGCCACGAGTTCGAGATCGAG<br>GGCAAG	pCS3703
ASD Clover Exon 3	CTGAGCCATCAGTCCGCCCTGAGCAAAGACCCCAA<br>CGAGAAGCGCGATCACATGGTCCTGCTGGAGTTCG<br>TGACCGCCGCCGGGATTACACATGGCATGGACGAG<br>CTGTACAAGGAT	pCS3704
ASD Clover Exon 4	GGCGAGGGCCGCCCCTACGAGGGCACCCAGACCGC<br>CAAGCTGAAAGTGACCAAGGGTGGCCCCCTGCCCT<br>TCGCCTGGGACATCCTGTCCCCTCAGTTCATGTACG<br>GCTCCAAGGCCTACGTGAAGCACCCCGCCGACATC<br>CCCGACTACTTGAAGCTGTCCTTCCCCGAGGGCTTC<br>AAGTGGGAGCGCGTGATGAACTTCGAGGACGGCG<br>GCGTGGTGACCGTGACCCAGGACTCCTCCCTGCAA<br>GACGGCGAGTTCATCTACAAAGTGAAGCTGCGCGG<br>CACCAACTTCCCCTCCGACGGCCCCGTAATGCAGA<br>AGAAGACCATGGGCTGGGAGGCCTCCTCCGAGCGG<br>ATGTACCCCGAGGACGGCGCCCTGAAGGGCGAGAT<br>CAAGCAGAGGCTGAAGCTGAAGGACGGCGGCCAC<br>TACGACGCTGAAGTCAAGACCACCTACAAGGCCAA<br>GAAGCCCGTGCAGCTGCCCGGAGCCTACAACGTCA<br>ACATCAAGTTGGACATCACCTCCCACAACGAGGAC<br>TACACCATCGTGGAACAGTACGAACGCGCCGAGGG<br>CCGCCACTCCACCGGCGGCATGGACGAGCTGTACA<br>AGTAA	pCS3703
TALE 1 Exon 1	ATGTCGCGGACCCGGCTCCCTTCCCCACCCGCACCC<br>AGCCCAGCGTTTTCGGCCGACTCGTTCTCAGACCTG<br>CTTAGGCAGTTCGACCCCTCACTGTTTAACACATCG<br>TTGTTCGACTCCCTTCCTCCGTTTGGGGCGCACCAT<br>ACGGAGGCGGCCACCGGGGAGTGGGATGAGGTGC<br>AGTCGGGATTGAGAGCTGCGGATGCACCACCCCCA<br>ACCATGCGGGTGGCCGTCACCGCTGCCCGACCGCC<br>GAGGGCGAAGCCCGCACCAAGGCGGAGGGCAGCG	This work

	CAACCGTCCGACGCAAGCCCCGCAGCGCAAGTAGA<br>TTTGAGAACTTTGGGATATTCACAGCAGCAGCAGG<br>AAAAGATCAAGCCCAAAGTGAGGTCGACAGTCGC<br>GCAGCATCACGAAGCGCTGGTGGGTCATGGGTTTA<br>CACATGCCCACATCGTAGCCTTGTCGCAGCACCCT<br>GCAGCCCTTGGCACGGTCGCCGTCAAGTACCAGGA<br>CATGATTGCGGCGTTGCCGGAAGCCACACATGAGG<br>CGATCGTCGGTGTGGGGAAACAGTGGAGCGGAGCC<br>CGAGCGCTTGAGGCCCTGTTGACGGTCGCGGGAGA<br>GCTGAGAGGGCCTCCCCTTCAGCTGGACACGGGCC<br>AGTTGCTGAAGATCGCGAAGCGGGGAGGAGTCAC<br>GGCGGTCGAGGCGGTGCACGCGTGGCGCAATGCGC<br>TCACGGGAGCACCCCTCAACCTGACCCCAGAGCAG<br>GTCGTGGCAATCGCCTCCAACATTGGCGGGAAACA<br>GGCACTCGAGACTGTCCAGCGCCTGCTTCCCGTGCT<br>GTGCCAAGCGCACGGCCTCACCCCAGAGCAGGTCG<br>TGGCGATCGCAAGCCACGACGGAGGAAAGCAAGC<br>CTTGGAAACAGTACAGAGGCTGTTGCCTGTGCTGT<br>GCCAAGCGCACGGCCTCACCCCAGAGCAG	
TALE 1 Exon 4	GCACTCGAGACTGTCCAGCGCCTGCTTCCCGTGCTG<br>TGCCAAGCGCACGGCTTAACCCCAGAGCAGGTCGT<br>GGCGATCGCAAGCCACGACGGAGGAAAGCAAGCC<br>TTGGAAACAGTACAGAGGCTGTTGCCTGTGCTGTG<br>CCAAGCGCACGGACTCACCCCAGAGCAGGTCGTGG<br>CCATTGCCTCGAATGGAGGGGGCAAACAGGCGTTG<br>GAAACCGTACAACGATTGCTGCCGGTGCTGTGCCA<br>AGCGCACGGCCTCACCCCAGAGCAGGTCGTGGCGA<br>TCGCAAGCCACGACGGAGGAAAGCAAGCCTTGGA<br>AACAGTACAGAGGCTGTTGCCTGTGCTGTGCCAAG<br>CGCACGGATTAACCCCAGAGCAGGTCGTGGCAATC<br>GCCTCCAACATTGGCGGGAAACAGGCACTCGAGAC<br>TGTCCAGCGCCTGCTTCCCGTGCTGTGCCAAGCGCA<br>CGGGCTCACCCCAGAGCAGGTCGTGGCGATCGCAA<br>GCCACGACGGAGGAAAGCAAGCCTTGGAAACAGT<br>ACAGAGGCTGTTGCCTGTGCTGTGCCAAGCGCACG<br>GACTCACCCCAGAGCAGGTCGTGGCCATTGCCTCG<br>AATGGAGGGGGCAAACAGGCGTTGGAAACCGTAC<br>AACGATTGCTGCCGGTGCTGTGCCAAGCGCACGGC<br>CTCACCCCAGAGCAGGTCGTGGCAATCGCCTCCAA<br>CATTGGCGGGAAACAGGCACTCGAGACTGTCCAGC<br>GCCTGCTTCCCGTGCTGTGCCAAGCGCACGGATTA<br>ACCCCAGAGCAGGTCGTGGCCATTGCCTCGAATGG<br>AGGGGGCAAACAGGCGTTGGAAACCGTACAACGA<br>TTGCTGCCGGTGCTGTGCCAAGCGCACGGACTCAC<br>GCCTGAGCAGGTAGTGGCTATTGCATCCAATATCG<br>GGGGCAGACCCGCACTGGAGTCAATCGTGGCCCAG<br>CTTTCGAGGCCGGACCCCGCGCTGGCCGCACTCAC<br>TAATGATCATCTTGTAGCGCTGGCCTGCCTCGGCGG<br>ACGACCCGCCTTGGATGCGGTGAAGAAGGGGCTCC	This work

	CGCACGCGCCTGCATTGATTAAGCGGACCAACAGA<br>AGGATTCCCGAGAGGACATCACATCGAGTGGCAGA<br>TCACGCGCAAGTGGTCCGCGTGCTCGGATTCTTCCA<br>GTGTCACTCCCACCCCGCACAAGCGTTCGATGACG<br>CCATGACTCAATTTGGTATGTCGAGACACGGACTG<br>CTGCAGCTCTTTCGTAGAGTCGGTGTCACAGAACTC<br>GAGGCCCGCTCGGGCACACTGCCTCCCGCCTCCCA<br>GCGGTGGGACAGGATTCTCCAAGCGAGCGGTATGA<br>AACGCGCGAAGCCTTCACCTACGTCAACTCAGACA<br>CCTGACCAGGCGAGCCTTCATGCGTTCGCAGACTC<br>GCTGGAGAGGGATTTGGACGCGCCCTCGCCCATGC<br>ATGAAGGGGACCAAACTCGCGCGTCAGCTAGCCCC<br>AAGAAGAAGAGAAAGGTGGAGGCCAGCGGTTCCG<br>GACGGGCTGACGCATTGGACGATTTTGATCTGGAT<br>ATGCTGGGAAGTGACGCCCTCGATGATTTTGACCTT<br>GACATGCTTGGTTCGGATGCCCTTGATGACTTTGAC<br>CTCGACATGCTCGGCAGTGACGCCCTTGATGATTTC<br>GACCTGGACATGCTGATTAACTCTAGAGGCAGTGG<br>AGAGGGCAGAGGAAGTCTGCTAACATGCGGTGAC<br>GTCGAGGAGAATCCTGGCCCAGTGAGCAAGGGCG<br>AGGAGCTGTTCACCGGGGTGGTGCCCATCCTGGTC<br>GAGCTGGACGGCGACGTAAACGGCCACAAGTTCAG<br>CGTCCGCGGCGAGGGCGAGGGCGATGCCACCAAC<br>GGCAAGCTGACCCTGAAGTTCATCTGCACCACCGG<br>CAAGCTGCCCGTGCCCTGGCCCACCCTCGTGACCA<br>CCTTCGGCTACGGCGTGGCCTGCTTCAGCCGCTACC<br>CCGACCACATGAAGCAGCACGACTTCTTCAAGTCC<br>GCCATGCCCGAAGGCTACGTCCAGGAGCGCACCAT<br>CTCTTTCAAGGACGACGGTACCTACAAGACCCGCG<br>CCGAGGTGAAGTTCGAGGGCGACACCCTGGTGAAC<br>CGCATCGAGCTGAAGGGCATCGACTTCAAGGAGGA<br>CGGCAACATCCTGGGGCACAAGCTGGAGTACAACT<br>TCAACAGCCACAACGTCTATATCACGGCCGACAAG<br>CAGAAGAACGGCATCAAGGCTAACTTCAAGATCCG<br>CCACAACGTTGAGGACGGCAGCGTGCAGCTCGCCG<br>ACCACTACCAGCAGAACACCCCCATCGGCGACGGC<br>CCCGTGCTGCTGCCCGACAACCACTACCTGAGCCA<br>TCAGTCCGCCCTGAGCAAAGACCCCAACGAGAAGC<br>GCGATCACATGGTCCTGCTGGAGTTCGTGACCGCC<br>GCCGGGATTACACATGGCATGGACGAGCTGTACAA<br>GTAA	
TALE 1 internal exon encoding A	GTCGTGGCAATCGCCTCCAACATTGGCGGAAACA<br>GGCACTCGAGACTGTCCAGCGCCTGCTTCCCGTGCT<br>GTGCCAAGCGCACGGATTAACCCCAGAGCAGGTCG<br>TGGCAATCGCCTCCAACATTGGCGGAAACAG	This work
TALE 1 internal exon encoding T	GTCGTGGCCATTGCCTCGAATGGAGGGGGCAAACA<br>GGCGTTGGAAACCGTACAACGATTGCTGCCGGTGC<br>TGTGCCAAGCGCACGGATTAACCCCAGAGCAGGTC<br>GTGGCAATCGCCTCCAACATTGGCGGGAAACAG	This work

TALE 1 internal exon encoding G	GTCGTGGCAATCGCGAGCAATAACGGCGGAAAAC<br>AGGCTTTGGAAACGGTGCAGAGGCTCCTTCCAGTG<br>CTGTGCCAAGCGCACGGATTAACCCCAGAGCAGGT<br>CGTGGCAATCGCCTCCAACATTGGCGGGAAACAG	This work
TALE 1 internal exon encoding C	GTCGTGGCCATTGCCTCGCACGACGGGGGCAAACA<br>GGCGTTGGAAACCGTACAACGATTGCTGCCGGTGC<br>TGTGCCAAGCGCACGGATTAACCCCAGAGCAGGTC<br>GTGGCAATCGCCTCCAACATTGGCGGGAAACAG	This work
TALE 2 Exon 1	ATGTCGCGGACCCGGCTCCCTTCCCCACCCGCACCC<br>AGCCCAGCGTTTTCGGCCGACTCGTTCTCAGACCTG<br>CTTAGGCAGTTCGACCCCTCACTGTTTAACACATCG<br>TTGTTCGACTCCCTTCCTCCGTTTGGGGCGCACCAT<br>ACGGAGGCGGCCACCGGGGAGTGGGATGAGGTGC<br>AGTCGGGATTGAGAGCTGCGGATGCACCACCCCCA<br>ACCATGCGGGTGGCCGTCACCGCTGCCCGACCGCC<br>GAGGGCGAAGCCCGCACCAAGGCGGAGGGCAGCG<br>CAACCGTCCGACGCAAGCCCCGCAGCGCAAGTAGA<br>TTTGAGAACTTTGGGATATTCACAGCAGCAGCAGG<br>AAAAGATCAAGCCCAAAGTGAGGTCGACAGTCGC<br>GCAGCATCACGAAGCGCTGGTGGGTCATGGGTTTA<br>CACATGCCCACATCGTAGCCTTGTCGCAGCACCCT<br>GCAGCCCTTGGCACGGTCGCCGTCAAGTACCAGGA<br>CATGATTGCGGCGTTGCCGGAAGCCACACATGAGG<br>CGATCGTCGGTGTGGGGAAACAGTGGAGCGGAGCC<br>CGAGCGCTTGAGGCCCTGTTGACGGTCGCGGGAGA<br>GCTGAGAGGGCCTCCCCTTCAGCTGGACACGGGCC<br>AGTTGCTGAAGATCGCGAAGCGGGGAGGAGTCAC<br>GGCGGTCGAGGCGGTGCACGCGTGGCGCAATGCGC<br>TCACGGGAGCACCCCTCAACCTGACCCCAGAGCAG<br>GTCGTGGCCATTGCCTCGAATGGAGGGGGCAAACA<br>GGCGTTGGAAACCGTACAACGATTGCTGCCGGTGC<br>TGTGCCAAGCGCACGGACTCACCCCAGAGCAGGTC<br>GTGGCCATTGCCTCGAATGGAGGGGGCAAACAGGC<br>GTTGGAAACCGTACAACGATTGCTGCCGGTGCTGT<br>GCCAAGCGCACGGCCTCACCCCAGAGCAGGTCGTG<br>GCCATTGCCTCGAATGGAGGGGGCAAACAGGCGTT<br>GGAAACCGTACAACGATTGCTGCCGGTGCTGTGCC<br>AAGCGCACGGATTAACCCCAGAGCAGGTCGTGGCA<br>ATCGCGAGCAATAACGGCGGAAAACAGGCTTTGGA<br>AACGGTGCAGAGGCTCCTTCCAGTGCTGTGCCAAG<br>CGCACGGCTTAACCCCAGAGCAGGTCGTGGCCATT<br>GCCTCGAATGGAGGGGGCAAACAGGCGTTGGAAA<br>CCGTACAACGATTGCTGCCGGTGCTGTGCCAAGCG<br>CACGGACTCACCCCAGAGCAG	This work

TALE 2 Exon 4	GCGTTGGAAACCGTACAACGATTGCTGCCGGTGCT<br>GTGCCAAGCGCACGGATTAACCCCAGAGCAGGTCG<br>TGGCCATTGCCTCGAATGGAGGGGGCAAACAGGCG<br>TTGGAAACCGTACAACGATTGCTGCCGGTGCTGTG<br>CCAAGCGCACGGGCTCACCCCAGAGCAGGTCGTGG<br>CGATCGCAAGCCACGACGGAGGAAAGCAAGCCTT<br>GGAAACAGTACAGAGGCTGTTGCCTGTGCTGTGCC<br>AAGCGCACGGACTCACCCCAGAGCAGGTCGTGGCC<br>ATTGCCTCGAATGGAGGGGGCAAACAGGCGTTGGA<br>AACCGTACAACGATTGCTGCCGGTGCTGTGCCAAG<br>CGCACGGCCTCACCCCAGAGCAGGTCGTGGCCATT<br>GCCTCGAATGGAGGGGGCAAACAGGCGTTGGAAA<br>CCGTACAACGATTGCTGCCGGTGCTGTGCCAAGCG<br>CACGGATTAACCCCAGAGCAGGTCGTGGCCATTGC<br>CTCGAATGGAGGGGGCAAACAGGCGTTGGAAACC<br>GTACAACGATTGCTGCCGGTGCTGTGCCAAGCGCA<br>CGGACTCACGCCTGAGCAGGTAGTGGCTATTGCAT<br>CCAATATCGGGGGCAGACCCGCACTGGAGTCAATC<br>GTGGCCCAGCTTTCGAGGCCGGACCCCGCGCTGGC<br>CGCACTCACTAATGATCATCTTGTAGCGCTGGCCTG<br>CCTCGGCGGACGACCCGCCTTGGATGCGGTGAAGA<br>AGGGGCTCCCGCACGCGCCTGCATTGATTAAGCGG<br>ACCAACAGAAGGATTCCCGAGAGGACATCACATCG<br>AGTGGCAGATCACGCGCAAGTGGTCCGCGTGCTCG<br>GATTCTTCCAGTGTCACTCCCACCCCGCACAAGCGT<br>TCGATGACGCCATGACTCAATTTGGTATGTCGAGA<br>CACGGACTGCTGCAGCTCTTTCGTAGAGTCGGTGTC<br>ACAGAACTCGAGGCCCGCTCGGGCACACTGCCTCC<br>CGCCTCCCAGCGGTGGGACAGGATTCTCCAAGCGA<br>GCGGTATGAAACGCGCGAAGCCTTCACCTACGTCA<br>ACTCAGACACCTGACCAGGCGAGCCTTCATGCGTT<br>CGCAGACTCGCTGGAGAGGGATTTGGACGCGCCCT<br>CGCCCATGCATGAAGGGGACCAAACTCGCGCGTCA<br>GCTAGCCCCAAGAAGAAGAGAAAGGTGGAGGCCA<br>GCGGTTCCGGACGGGCTGACGCATTGGACGATTTT<br>GATCTGGATATGCTGGGAAGTGACGCCCTCGATGA<br>TTTTGACCTTGACATGCTTGGTTCGGATGCCCTTGA<br>TGACTTTGACCTCGACATGCTCGGCAGTGACGCCCT<br>TGATGATTTCGACCTGGACATGCTGATTAACTCTAG<br>AGGCAGTGGAGAGGGCAGAGGAAGTCTGCTAACA<br>TGCGGTGACGTCGAGGAGAATCCTGGCCCAGTGAG<br>CAAGGGCGAGGAGCTGTTCACCGGGGTGGTGCCCA<br>TCCTGGTCGAGCTGGACGGCGACGTAAACGGCCAC<br>AAGTTCAGCGTCCGCGGCGAGGGCGAGGGCGATGC<br>CACCAACGGCAAGCTGACCCTGAAGTTCATCTGCA<br>CCACCGGCAAGCTGCCCGTGCCCTGGCCCACCCTC<br>GTGACCACCTTCGGCTACGGCGTGGCCTGCTTCAG<br>CCGCTACCCCGACCACATGAAGCAGCACGACTTCT<br>TCAAGTCCGCCATGCCCGAAGGCTACGTCCAGGAG	This work

	CGCACCATCTCTTTCAAGGACGACGGTACCTACAAGACCCGCGCCGAGGTGAAGTTCGAGGGCGACACCCTGGTGAACCGCATCGAGCTGAAGGGCATCGACTTCAAGGAGGACGGCAACATCCTGGGGCACAAGCTGGAGTACAACTTCAACAGCCACAACGTCTATATCACGGCCGACAAGCAGAAGAACGGCATCAAGGCTAACTTCAAGATCCGCCACAACGTTGAGGACGGCAGCGTGCAGCTCGCCGACCACTACCAGCAGAACACCCCCATCGGCGACGGCCCCGTGCTGCTGCCCGACAACCACTACCTGAGCCATCAGTCCGCCCTGAGCAAAGACCCCAACGAGAAGCGCGATCACATGGTCCTGCTGGAGTTCGTGACCGCCGCCGGGATTACACATGGCATGGACGAGCTGTACAAGTAA	
TALE 2 internal exon encoding AA	GTCGTGGCAATCGCCTCCAACATTGGCGGGAAACAGGCACTCGAGACTGTCCAGCGCCTGCTTCCCGTGCTGTGCCAAGCGCACGGCCTCACCCCAGAGCAGGTCGTGGCAATCGCCTCCAACATTGGCGGGAAACAG	This work
TALE 2 internal exon encoding TT	GTCGTGGCCATTGCCTCGAATGGAGGGGGCAAACAGGCGTTGGAAACCGTACAACGATTGCTGCCGGTGCTGTGCCAAGCGCACGGCCTCACCCCAGAGCAGGTCGTGGCCATTGCCTCGAATGGAGGGGGCAAACAG	This work
TALE 2 internal exon encoding GG	GTCGTGGCAATCGCGAGCAATAACGGCGGAAAACAGGCTTTGGAAACGGTGCAGAGGCTCCTTCCAGTGCTGTGCCAAGCGCACGGCCTCACCCCAGAGCAGGTCGTGGCAATCGCGAGCAATAACGGCGGAAAACAG	This work
TALE 2 internal exon encoding CC	GTCGTGGCGATCGCAAGCCACGACGGAGGAAAGCAAGCCTTGGAAACAGTACAGAGGCTGTTGCCTGTGCTGTGCCAAGCGCACGGCCTCACCCCAGAGCAGGTCGTGGCGATCGCAAGCCACGACGGAGGAAAGCAA	This work

Supplementary References

1. Culler, S. J., Hoff, K. G., Voelker, R. B., Berglund, J. A. & Smolke, C. D. Functional selection and systematic analysis of intronic splicing elements identify active sequence motifs and associated splicing factors. *Nucleic Acids Res.* **38**, 5152–5165 (2010).
2. Gromak, N. *et al.* The PTB interacting protein raver1 regulates α-tropomyosin alternative splicing. *EMBO J.* **22**, 6356–6364 (2003).
3. Feng, H., Qin, Z. & Zhang, X. Opportunities and methods for studying alternative splicing in cancer with RNA-Seq. *Cancer Lett.* **340**, 179–191 (2013).
