# Supplementary material for: Programmable mutually exclusive alternative splicing for generating RNA and protein diversity
Source: Nat Commun. 2019 Jun 17;10:2673. doi: 10.1038/s41467-019-10403-w (PMC6572816; doi:10.1038/s41467-019-10403-w)
Supplement: Supplementary file 3 — Description of Additional Supplementary Files [file 41467_2019_10403_MOESM3_ESM.docx]

**Title:** Supplementary Data 1
**Description:** Isoform abundances and sequence information from long-read sequencing of ASDs.
