# Supplementary material for: Programmable mutually exclusive alternative splicing for generating RNA and protein diversity
Source: Nat Commun. 2019 Jun 17;10:2673. doi: 10.1038/s41467-019-10403-w (PMC6572816; doi:10.1038/s41467-019-10403-w)
Supplement: Supplementary file 5 — Reporting Summary [file 41467_2019_10403_MOESM5_ESM.pdf]

nature research

Corresponding author(s): Christina D. Smolke

Last updated by author(s): 4/26/2019

Reporting Summary

Nature Research wishes to improve the reproducibility of the work that we publish. This form provides structure for consistency and transparency in reporting. For further information on Nature Research policies, see Authors & Referees and the Editorial Policy Checklist.

Statistics

For all statistical analyses, confirm that the following items are present in the figure legend, table legend, main text, or Methods section.

n/a	Confirmed	
☐	☒	The exact sample size (*n*) for each experimental group/condition, given as a discrete number and unit of measurement
☐	☒	A statement on whether measurements were taken from distinct samples or whether the same sample was measured repeatedly
☒	☐	The statistical test(s) used AND whether they are one- or two-sided<br>*Only common tests should be described solely by name; describe more complex techniques in the Methods section.*
☒	☐	A description of all covariates tested
☒	☐	A description of any assumptions or corrections, such as tests of normality and adjustment for multiple comparisons
☐	☒	A full description of the statistical parameters including central tendency (e.g. means) or other basic estimates (e.g. regression coefficient) AND variation (e.g. standard deviation) or associated estimates of uncertainty (e.g. confidence intervals)
☒	☐	For null hypothesis testing, the test statistic (e.g. *F*, *t*, *r*) with confidence intervals, effect sizes, degrees of freedom and *P* value noted<br>*Give P values as exact values whenever suitable.*
☒	☐	For Bayesian analysis, information on the choice of priors and Markov chain Monte Carlo settings
☒	☐	For hierarchical and complex designs, identification of the appropriate level for tests and full reporting of outcomes
☒	☐	Estimates of effect sizes (e.g. Cohen's *d*, Pearson's *r*), indicating how they were calculated

Our web collection on statistics for biologists contains articles on many of the points above.

Software and code

Policy information about availability of computer code

Data collection	The MACSQuant VYB flow cytometer and the Pacific Biosciences RS II instrument were used to collect data in this study.
Data analysis	FlowJo 7, Excel, and the Pacific Biosciences SMRT Analysis software (v2.3.0) were used to analyze the data in this study.

For manuscripts utilizing custom algorithms or software that are central to the research but not yet described in published literature, software must be made available to editors/reviewers. We strongly encourage code deposition in a community repository (e.g. GitHub). See the Nature Research guidelines for submitting code & software for further information.

Data

Policy information about availability of data

All manuscripts must include a data availability statement. This statement should provide the following information, where applicable:

- Accession codes, unique identifiers, or web links for publicly available datasets
- A list of figures that have associated raw data
- A description of any restrictions on data availability

The authors declare that all data supporting the findings of this study are available within the paper and its supplementary information files. Source data underlying Figs. 2d-g, 3b-d, 4b-c, 5b-c, 6b-c, and Supplementary Figs. 1b-c, 2b-c, 4b-c, 5a-c, 7a-c, 9a-f, 11b-c, 12a-b, 13a-f, and 14a-b are provided in the Source Data file. Long-read sequencing data from the splicing devices are available in the Supplementary Data 1 file.

Field-specific reporting

Please select the one below that is the best fit for your research. If you are not sure, read the appropriate sections before making your selection.

☒ Life sciences ☐ Behavioural & social sciences ☐ Ecological, evolutionary & environmental sciences

For a reference copy of the document with all sections, see nature.com/documents/nr-reporting-summary-flat.pdf

Life sciences study design

All studies must disclose on these points even when the disclosure is negative.

Sample size	Sample sizes of n = 2 or n = 3 per experiment were chosen to be cost efficient.
Data exclusions	No fluorescence data were excluded from the study. For the long-read sequencing experiments, transcripts accounting for less than 1% of the total number of reads were excluded as they were pre-determined to be sequencing artifacts.
Replication	Flow cytometry experiments were successfully reproduced by the co-authors of the paper. The long-read sequencing experiments were conducted with sample sizes of n = 2 or n = 3 to confirm precision across replicate samples.
Randomization	Cultured cells were randomly allocated on experimental plates that were run on the flow cytometer or sequencer.
Blinding	Investigators were not blinded to experiments. Blinding was not necessary as fluorescence levels and transcript abundance values were measured using instruments that were not expected to be affected by investigator bias.

Reporting for specific materials, systems and methods

We require information from authors about some types of materials, experimental systems and methods used in many studies. Here, indicate whether each material, system or method listed is relevant to your study. If you are not sure if a list item applies to your research, read the appropriate section before selecting a response.

Materials & experimental systems

n/a	Involved in the study	
☒	☐	Antibodies
☐	☒	Eukaryotic cell lines
☒	☐	Palaeontology
☒	☐	Animals and other organisms
☒	☐	Human research participants
☒	☐	Clinical data

Methods

n/a	Involved in the study	
☒	☐	ChIP-seq
☐	☒	Flow cytometry
☒	☐	MRI-based neuroimaging

Eukaryotic cell lines

Policy information about cell lines

Cell line source(s)	HEK-293T cells were acquired from ATCC. In the supplementary information, data from HeLa cells (a generous gift from the James Chen Laboratory, Stanford, CA), CHO-K1 cells (acquired from ATCC), and U2OS cells (a generous gift from the Katrin Chua Laboratory, Stanford, CA) are presented.
Authentication	Cells were visually authenticated using a Zeiss Axiovert 200 M Inverted Microscope.
Mycoplasma contamination	We employed good aseptic technique to reduce the risk of mycoplasma contamination.
Commonly misidentified lines (See ICLAC register)	None of the cell lines presented are listed in the ICLAC database.

Flow Cytometry

Plots

Confirm that:

☒ The axis labels state the marker and fluorochrome used (e.g. CD4-FITC).

☒ The axis scales are clearly visible. Include numbers along axes only for bottom left plot of group (a 'group' is an analysis of identical markers).

☒ All plots are contour plots with outliers or pseudocolor plots.

☒ A numerical value for number of cells or percentage (with statistics) is provided.

Methodology

Sample preparation	All transfected cell cultures were suspended in media (DMEM+10% FBS).
Instrument	The MACSQuant VYB instrument was used for flow cytometry in this study.
Software	The MACSQuantify software (v 2.8) was used to collect the data. Data were analyzed using FlowJo 7 and Excel.
Cell population abundance	The relevant cell populations were abundant in the post-sort fractions.
Gating strategy	Viability was gated by SSC-A vs. FSC-A and then singlets were gated by FSC-H vs. FSC-A. In cells harboring the BFP transfection marker, singlets were further gated for BFP-positive cells by SSC-A vs. V1-A. mCherry-positive cells were gated by SSC-A vs. Y2-A and Clover-positive cells were gated by SSC-A vs. B1-A.

☒ Tick this box to confirm that a figure exemplifying the gating strategy is provided in the Supplementary Information.
